# Supplementary material for: A VersaTile Approach to Reprogram the Specificity of the R2-Type Tailocin Towards Different Serotypes of Escherichia coli and Klebsiella pneumoniae
Source: Antibiotics (Basel). 2025 Jan 18;14(1):104. doi: 10.3390/antibiotics14010104 (PMC11762384; doi:10.3390/antibiotics14010104)
Supplement: Supplementary file 1 [file antibiotics-14-00104-s001.zip › antibiotics-3333159-supplementary.pdf]

These supplementary materials belong to:

A VersaTile approach to reprogram and broaden the specificity of R2-tailocins towards *E. coli* and *K. pneumoniae* serotypes

Dorien Dams†, Célia Pas†, Agnieszka Latka, Zuzanna Drulis-Kawa, Lars Fieseler, Yves Briers*

* Correspondence: yves.briers@ugent.be; Tel.:+32-09-243-24-53

† These authors contributed equally to this work.

Table of Contents

Supplementary Figures and Tables 3

Supplementary Table S1. Amino acid sequences of chimeric receptor-binding proteins (RBPs)... 3

Supplementary Figure S1. AlphaFold3 predicted receptor-binding protein (RBP) structures...... 10

Supplementary Figure S2. An overview of the protein concentrations for each biological replicate of produced engineered R2 tailocin. 11

Supplementary Figure S3. Results of the spot assays of engineered R2 tailocins. 13

Supplementary Figure S4. Survival and growth inhibition assays of the different engineered R2 tailocins.. 19

Supplementary Figure S5. 24-hour growth curves of the growth inhibition assay...................... 26

Supplementary Figure S6. Schematic overview and experimental results of R2 tailocin R2-K11 purified by two different methods. 27

Supplementary Table S2. List of all bacterial strains and their origin used in this research......... 28

Supplementary Figure S7. Map of the pVTE cloning vector used in the VersaTile cloning step to construct tiles. 29

Supplementary Figure S8. Map of pVTD27 shuttle expression vector.. 30

Supplementary Figure S9. Map of pVTD29 shuttle expression vector... 30

Supplementary Table S3. Primers for tile preparation... 31

Supplementary Table S4. Components and conditions for tile sequence amplification for the VersaTile cloning step using Phusion polymerase.. 33

Supplementary Table S5. Components and conditions for the restriction-ligation reaction of the VersaTile cloning step.. 33

Supplementary Table S6. Components and conditions for the restriction-ligation reaction of the VersaTile assembly step 34

Supplementary Table S7. Overview of the different wild-type and engineered R2 tailocins that were produced in this work.. 35

Supplementary Figure S10. Statistical analysis of the survival assay. 39

Supplementary Figures and Tables

Supplementary Table S1. Amino acid sequences of chimeric receptor-binding proteins (RBPs).

Amino acid protein sequence in FASTA format
>Tail_fiber_R2prf15<br>MTTNTPKYGGLLTDIGAAALATASAAGKKWQPTHMLIGDAGGAPGDTPDPLPSAAQKSLINQRHRAQLNRLFVSDKNANTLVAEVVLPVEVG<br>GFWIREIGLQDADGKFVAVSNCPPSYKAAMESGSARTQTIRVNIALSGLENVQLLIDNGIIYATQDWVKEKVAADFKGRKILAGNGLLGGGD<br>LSADRSIGLAPSGVTAGSYRSVTVNANGVVTQGSNPTTLAGYAIGDAYTKADTDGKLAQKANKATTLAGYGITDALRVDGNAVSSSRLAAPR<br>SLAASGDASWSVTFDGSANVSAPLSLSATGVAAGSYPKVTVDTKGRVTAGMALAATDIPGLDASKLVSGVLAEQRLPVFARGLATAVSNSSD<br>PNTATVPLMLTNHANGPVAGRYFYIQSMFYPDQNGNASQIATSYNATSEMYVRVSYAANPSIREWLPWQRCDIGGSFTKEADGELPGGVNLD<br>SMVTSGWWSQSFTAQAASGANYPIVRAGLLHVYAASSNFIYQTYQAYDGESFYFRCRHSNTWFPWRRMWHGGDFNPSDYLLKSGFYWNALPG<br>KPATFPPSAHNHDVGQLTSGILPLARGGVGSNTAAGARSTIGAGVPATASLGASGWWRDNDTGLIRQWGQVTCPADADASITFPIPFPTLCL<br>GGYANQTSAFHPGTDASTGFRGATTTTAVIRNGYFAQAVLSWEAFGR<br>>Chaperone_R2prf16<br>MGAGHLPRRCRCFDYVPDSFPYAMPRRICESDECFPSGNGCQYRFPWSDYHYRGDSQWLLCSGGSFMGGIWTMKGEYYFSPSQVAFYPASLR<br>EVYEYAGCWPVDGEWVSAELHEQLMNEQAAGRAISSDVNGNPVAIERPPLSRQQRSTHERRWRDSQLLATDGLVVRHRDQLETGKETTLLPV<br>QYHELMSYRASLRDWPEEPLFPDSGGRPSVPDWLRRYVTP
>Tailspike_R2-WT-VT_R2prf15<br>MTTNTPKYGGLLTDIGAAALATASAAGKKWQPTHMLIGDAGGAPGDTPDPLPSAAQKSLINQRHRAQLNRLFVSDKNANTLVAEVVLPVEVG<br>GFWIREIGLQDADGKFVAVSNCPPSYKAAMESGSARTQTIRVNIALSGLENVQLLIDNGIIYATQDWVKEKGSVAADFKGRKILAGNGLLGG<br>GDLSADRSIGLAPSGVTAGSYRSVTVNANGVVTQGSNPTTLAGYAIGDAYTKADTDGKLAQKANKATTLAGYGITDALRVDGNAVSSSRLAA<br>PRSLAASGDASWSVTFDGSANVSAPLSLSATGVAAGSYPKVTVDTKGRVTAGMALAATDIPGLDASKLVSGVLAEQRLPVFARGLATAVSNS<br>SDPNTATVPLMLTNHANGPVAGRYFYIQSMFYPDQNGNASQIATSYNATSEMYVRVSYAANPSIREWLPWQRCDIGGSFTKEADGELPGGVN<br>LDSMVTSGWWSQSFTAQAASGANYPIVRAGLLHVYAASSNFIYQTYQAYDGESFYFRCRHSNTWFPWRRMWHGGDFNPSDYLLKSGFYWNAL<br>PGKPATFPPSAHNHDVGQLTSGILPLARGGVGSNTAAGARSTIGAGVPATASLGASGWWRDNDTGLIRQWGQVTCPADADASITFPIPFPTL<br>CLGGYANQTSAFHPGTDASTGFRGATTTTAVIRNGYFAQAVLSWEAFGR<br>>Chaperone_R2prf16<br>MGAGHLPRRCRCFDYVPDSFPYAMPRRICESDECFPSGNGCQYRFPWSDYHYRGDSQWLLCSGGSFMGGIWTMKGEYYFSPSQVAFYPASLR<br>EVYEYAGCWPVDGEWVSAELHEQLMNEQAAGRAISSDVNGNPVAIERPPLSRQQRSTHERRWRDSQLLATDGLVVRHRDQLETGKETTLLPV<br>QYHELMSYRASLRDWPEEPLFPDSGGRPSVPDWLRRYVTP
>Tailspike_R2-O26<br>MTTNTPKYGGLLTDIGAAALATASAAGKKWQPTHMLIGDAGGAPGDTPDPLPSAAQKSLINQRHRAQLNRLFVSDKNANTLVAEVVLPVEVG<br>GFWIREIGLQDADGKFVAVSNCPPSYKAAMESGSARTQTIRVNIALSGLENVQLLIDNGIIYATQDWVKEKGSLLSEDHSLGDSLVMHNPQF<br>VDSTSTILSKKLTIDLRTLTDYGFAEGNTGAQNKAAFQKAIDDATLPTEIVIPEGAFIVDPGITIKNTVTMIRGAGAYQSRIFSTGTAAPII<br>TQQDGVITFCEFRDFGLDGNGYAANGISLTEANHIKIENIDVVNTNNNAILVNGYSIDIIGCRLFQNAGNGINVGGYCNNINIINSRIYGNG<br>AGGVLLTPAYAEGGMSVRVNGNNIEQNKFYGLLAYGVKGLNLDANYWERNGEIGYPYSVPESITVRADIHLIANNFTLIPDLSKINDTVSIR<br>GNQQTAIGYASALPNQDGFIFTNYAKNLTIENNQLLDASKVNNLLAMYHNNLSSKVTDRLYLANNTVNSIGYVGSYDPATQNPDTAHLIDIA<br>NRELTANYLDRNMLLWTAASGTTGTLIKTQNIYAGNYSFLVTTGDRVWGRTIDLNKSPELKGKFVWFGAWVNDQGSASKLMFIINGAGQTDS<br>TAPLAGNGKWSYVSCGVYIYETDTAINVGIRNYGSGNVLINSPSLCAYGMPSNALQVEKTTFYLSSVPTSGFWDIGERVINSAPASGQPKAW<br>TCNIPGGPGTYSFLSEGNF
>Tailspike_R2-O103-a<br>MTTNTPKYGGLLTDIGAAALATASAAGKKWQPTHMLIGDAGGAPGDTPDPLPSAAQKSLINQRHRAQLNRLFVSDKNANTLVAEVVLPVEVG<br>GFWIREIGLQDADGKFVAVSNCPPSYKAAMESGSARTQTIRVNIALSGLENVQLLIDNGIIYATQDWVKEKGSSLVSLVRDTTQDIKHKADI<br>LLDYLDRQEQGLGQVQEFVESSTILNIPSLRSLLPSSEGQVVQVLSSYSTAATQLHLGGGQFQAVNNLQAEPDDGGIVVHSTHPTLVWKRIN<br>FTAYDMQFWGVVADASTDNAAAITRATQYARRNRIVLEAPAGSIHTSEMVPLYDNMGIRGHGRAENTVFYKTTNNGFNYTKNGVTVLTVDAL<br>AGFVPKEWDLADYEIKSFCVHAQLIGCMFRRLGLSAENVATTRPQYGLHIGKAATAVVRQCTFEGGYIGIKGYNVFSSVFESVAAVQFNGYG

YAGVSISDYRNSTLYNSGTSLDMRIVQVRGYQFGFELDRLQYSTLIDCTAEEIVPMAGETTSYAFSMYDPFCITMLNCATEFVKGGQIRVLG
FANPSFRPQLKVTGFIAIDQQNPAALTPIYLVDNGGTTGMNVVIEASELSRNTALSNNGQPSVQGAGAKVILIGCSGEDWVASGSGVFTRLA

>Tailspike_R2-O103-b

MTTNTPKYGGLLTDIGAAALATASAAGKKWQPTHMLIGDAGGAPGDTPDPLPSAAQKSLINQRHRAQLNRLFVSDKNANTLVAEVVLPVEVG
GFWIREIGLQDADGKFVAVSNCPPSYKAAMESGSARTQTIRVNIALSGLENVQLLIDNGIIYATQDWVKEKLGSMSLVSLVRDTTQDIKHKA
DILLDYLDRQEQGLGQVQEFVESSTILNIPSLRSLLPSSEGQVVQVLSSYSTAATQLHLGGGQFQAVNNLQAEPDDGGIVVHSTHPTLVWKR
INFTAYDMQFWGVVADASTDNAAAITRATQYARRNRIVLEAPAGSIHTSEMVPLYDNMGIRGHGRAENTVFYKTTNNGFNYTKNGVTVLTVD
ALAGFVPKEWDLADYEIKSFCVHAQLIGCMFRRLGLSAENVATTRPQYGLHIGKAATAVVRQCTFEGGYIGIKGYNVFSSVFESVAAVQFNG
YGYAGVSISDYRNSTLYNSGTSLDMRIVQVRGYQFGFELDRLQYSTLIDCTAEEIVPMAGETTSYAFSMYDPFCITMLNCATEFVKGGQIRV
LGFANPSFRPQLKVTGFIAIDQQNPAALTPIYLVDNGGTTGMNVVIEASELSRNTALSNNGQPSVQGAGAKVILIGCSGEDWVASGSGVFTR
LA

>Tailspike_R2-O104-a

MTTNTPKYGGLLTDIGAAALATASAAGKKWQPTHMLIGDAGGAPGDTPDPLPSAAQKSLINQRHRAQLNRLFVSDKNANTLVAEVVLPVEVG
GFWIREIGLQDADGKFVAVSNCPPSYKAAMESGSARTQTIRVNIALSGLENVQLLIDNGIIYATQDWVKEKGSLQGSAGVIIVGGAILECSS
VEQAQSILGLTDGKKVRTYYYNVPVVTDWVFTTEKPQSPIFYISAVGGYLILMTPNFASAGIIEGEYVPLNAAHNRNVIQKLVRDTRFSKFE
YGATGTFYVLGSIHPLRDNIEIHHEAGVTVMGRYDDPTVGPEIMGLNSGGMWSFAHFLNPDATVWDGNNYTITAVMKNVTYILNGKIGTEYN
SIHSKPHNNNPIGFFTCENCSVVGSGGVIGSDHRGINVDGAGDNCRIDIGYVTGTSNNPLQMKINAERYAFVKVGAVYGVKFDGGQTNAVVW
CSGGRIDVEVGNYRWDGVNKPILAYGNNCQEITLKAGIISGVSQLMRQYNTPVARVLSAWVNNTDSLINKAEDTDKAGVTKISEIRGVNADD
AVLTSVFYDETKLSTQEVVIICDNNFKKASSSFVYFKGKTSSGAITSWYSQNNIDPSNITDFSDWNIKIGQSGLLSISGLSFSYKINRRFQF
LSVQFVDSGGTERVITIPIVMTLATTTTVSYIAGTSNLSVTRVGDTLSAVLSAGSLRFAKEHN

>Tailspike_R2-O111-a

MTTNTPKYGGLLTDIGAAALATASAAGKKWQPTHMLIGDAGGAPGDTPDPLPSAAQKSLINQRHRAQLNRLFVSDKNANTLVAEVVLPVEVG
GFWIREIGLQDADGKFVAVSNCPPSYKAAMESGSARTQTIRVNIALSGLENVQLLIDNGIIYATQDWVKEKGSQLRQELAGPNGYLLIPSMD
QHIKIQQWREEGDIRGWGAIDGEFNDAAVSAALDSESPSVKLGGVGFVSKLRSPINHKSNKVMHSGSLNFQFDGGTQQEKSGILMANISNAK
VIDVDITGTLDGGIRGYGGSNIVIDGVNVHDIGISTLSGECGIGIWFGDYANYDVQTDGLLIQNCNIKNIGGVGIQRGDGILVYNAKNFKVR
YNTIITTNRMAIAAGSDTRQFEIHGNYIGDTLLAGIDIEPDEGYTASNFKIYNNNIIGFAARYFTQGSGVGQTFGIDTHANTSYGKVYKNTL
SAGQYGTEAFHIGNHADEIEVTDNNLIGGAVVIPLFIKTYDGSGSKHIKINRNRAKGTCKSFADVAMSEDVYISENVFSGNSSNDSYFLRAS
TIAGLNVDYNRSSNTTNFIKSGDAGNTSSVKVTNNNASTLSDGIDMLTSGSMTDFIASGNTILCPASNKGISLEVAGSGSISDISLRGNIIY
NATTKIYVSPAATGWDMLTKNTRFDLSGVQNGTQLFELSRNRVTQFLNNAWYDG

>Tailspike_R2-O145-a

MTTNTPKYGGLLTDIGAAALATASAAGKKWQPTHMLIGDAGGAPGDTPDPLPSAAQKSLINQRHRAQLNRLFVSDKNANTLVAEVVLPVEVG
GFWIREIGLQDADGKFVAVSNCPPSYKAAMESGSARTQTIRVNIALSGLENVQLLIDNGIIYATQDWVKEKGSFPQQLSQLGLYVNDDSKGD
AMIGVKQPITGSIHRTQHDVNYERISAFDLGVKGDGVTDDVTAIREALITAATARRSIHFPDGVYLCSDFFSIPSHSRIYCDPGAVFKLTGS
TSLGGFAVTGLNNQVQTELCEDVVTYNMTLDCNKISGENGMNGVTCRNIRHYNLTVINTLHNAESRGGRAFQCEGGISSDVHVFNFVIKNCS
IGVNYQGLPDASQKVAAFSFHGVAMENVDVPFCIYSQISNPESNTPEIMSVAAFDVSCHNCGKITGGYGDQLGAGIICGDRGYGLFIDGIRV
VNESSYGGIGAFIRGQMFGVSINNADIYCIYAVSVIDHNKIGFDDAAVNTVPSSVRVRNLNVSSNLDFIVRAPDSSAIGNSTVDVSVNITRA
SLNQLFDSGAGGSASAVINISDSLTGKSSGFRTLKNIFDAGNSIGLLSRYDAGGSWVPIDGSGAGIAILLTGQQKYTKAGEVVHFYMNIIYP
VNDNTNDAVISGLPFNSVPFVSQFAGAASIAIKSSSMLSTVGILPGGDKLKFYGTTGAPLTNRDMSGVSVAVFGSYISE

>Tailspike_R2-O145-b

MTTNTPKYGGLLTDIGAAALATASAAGKKWQPTHMLIGDAGGAPGDTPDPLPSAAQKSLINQRHRAQLNRLFVSDKNANTLVAEVVLPVEVG
GFWIREIGLQDADGKFVAVSNCPPSYKAAMESGSARTQTIRVNIALSGLENVQLLIDNGIIYATQDWVKEKLGSFPQQLSQLGLYVNDDSKG
DAMIGVKQPITGSIHRTQHDVNYERISAFDLGVKGDGVTDDVTAIREALITAATARRSIHFPDGVYLCSDFFSIPSHSRIYCDPGAVFKLTG
STSLGGFAVTGLNNQVQTELCEDVVTYNMTLDCNKISGENGMNGVTCRNIRHYNLTVINTLHNAESRGGRAFQCEGGISSDVHVFNFVIKNC
SIGVNYQGLPDASQKVAAFSFHGVAMENVDVPFCIYSQISNPESNTPEIMSVAAFDVSCHNCGKITGGYGDQLGAGIICGDRGYGLFIDGIR
VVNESSYGGIGAFIRGQMFGVSINNADIYCIYAVSVIDHNKIGFDDAAVNTVPSSVRVRNLNVSSNLDFIVRAPDSSAIGNSTVDVSVNITR
ASLNQLFDSGAGGSASAVINISDSLTGKSSGFRTLKNIFDAGNSIGLLSRYDAGGSWVPIDGSGAGIAILLTGQQKYTKAGEVVHFYMNIIY
PVNDNTNDAVISGLPFNSVPFVSQFAGAASIAIKSSSMLSTVGILPGGDKLKFYGTTGAPLTNRDMSGVSVAVFGSYISE

>Tailspike_R2-O146-a

MTTNTPKYGGLLTDIGAAALATASAAGKKWQPTHMLIGDAGGAPGDTPDPLPSAAQKSLINQRHRAQLNRLFVSDKNANTLVAEVVLPVEVG
GFWIREIGLQDADGKFVAVSNCPPSYKAAMESGSARTQTIRVNIALSGLENVQLLIDNGIIYATQDWVKEKGSLAKPTGASYIGIPKGGFLS
DVLYYSTPKEFGGVGDGITDDTAAIVAAVNTGLPLVLCGGKWRITSTIELPDCYAVDARCSHIIADTGFQPVFLFKGKNYGLHYAGGLVTGT
ASSFMYIEGETDVPTTSNQYAKLVELYGVNISSKTIITSLIMKKAVRQIFIDSCFFYSKNGIEAEGKTVEITIDKSIIYGSTADTDSFGIKL
SSTGGTRFYNEGWHISDSTIDNFANTLDLADIFVFTVSKGYVACNATTAYAVTIRPPTTTTHTREIQFDGVLGGKIRFLDTVSGQTIFHTKI
SGEITNCKQGTCLSIGANAAGIDIRSLRVTSSPGCTLSSVANGSANINYSDISTDSSLAAGIIFNGEAGGNCSISDYLYSGTGEALSLARPV
KLSNVPTVGSETLNWSIKYDYVSASKDVAVGAYINQLQMTIARYSKIEININLSYSGGAESNAQAIGITAPTGVVLPNGTNSMLFTLPATAG
SLTMSIVATTTLDFKNLSFSIDNRAGNQLRILAGSHLSVSLAN

>Tailspike_R2-O157-a

MTTNTPKYGGLLTDIGAAALATASAAGKKWQPTHMLIGDAGGAPGDTPDPLPSAAQKSLINQRHRAQLNRLFVSDKNANTLVAEVVLPVEVG
GFWIREIGLQDADGKFVAVSNCPPSYKAAMESGSARTQTIRVNIALSGLENVQLLIDNGIIYATQDWVKEKGSQLAANDGFKFIGQCPDILT
LRTIEPEKNGQRITLRQHTIGTGLGGGVFRAVLDGTGYTDDDGVVIKTAGGSVWLRVNADKVNPFMFGATGVADDTAALQKMLECGRAAELG
TNVWKASNLELNNKSCSLSGSGLHVSRIEQISGATGALLTITQDCSLIYLSDCGLYGDGITAGTSGVTMETGNPGGAPSYPFNTAPDVRRDL
YISNVHITGFDELGFDYPETNFSVSTHGLFIRNIKKTGAKIGTTDFTWTNLQIDTCGQECLVLDGAGNCRIIGAKLIWAGSENETPYSGLRI
SNSQNVNMTGVELQDCAYDGLYIKNSTVAISGLNTNRNSASSNLSYHNMVFENSIVTVDGYVCRNYAATSLYDLNSQAGNVRCIGSDSTVLI
NGIYESEVNSERLMGDNNLIQPYSGDLIINGLKNYYTYTGSVKNNIPTFDGVVTTATYVSAPSILGQGNMLKLTQSNKDKLLFSDKVSRHGC
TIGLVLIPSFTGATTMTAFTLGSGYSPSGNSAVMQFIVNSSGVQTIAILLSGDGITQTLTSDLTTEQALASGGVYHFAMGFAPGRLWWSIID
INTGRRIRRAYRQPDLHAAFNSIFNSGTSSITAFSGPLAGDIACEGAGSHVYVGGFSSESDYAASRMYGLFTPVDLDKQYSFRTLNGNI

>Tailspike_R2-K1

MTTNTPKYGGLLTDIGAAALATASAAGKKWQPTHMLIGDAGGAPGDTPDPLPSAAQKSLINQRHRAQLNRLFVSDKNANTLVAEVVLPVEVG
GFWIREIGLQDADGKFVAVSNCPPSYKAAMESGSARTQTIRVNIALSGLENVQLLIDNGIIYATQDWVKEKGSAKGDGVTDDTAALTSALND
TPVGQKINGNGKTYKVTSLPDISRFINTRFVYERIPGQPLYYASEEFVQGELFKITDTPYYNAWPQDKAFVYENVIYAPYMGSDRHGVSRLH
VSWVKSGDDGQTWSTPEWLTDLHPDYPTVNYHCMSMGVCRNRLFAMIETRTLAKNALTNCALWDRPMSRSLHLTGGITKAANQRYATIHVPD
HGLFVGDFVNFSNSAVTGVSGDMTVATVIDKDNFTVLTPNQQTSDLNNAGKNWHMGTSFHKSPWRKTDLGLIPSVTEVHSFATIDNNGFAMG
YHQGDVAPREVGLFYFPDAFNSPSNYVRRQIPSEYEPDASEPCIKYYDGVLYLITRGTRGDRLGSSLHRSRDIGQTWESLRFPHNVHHTTLP
FAKVGDDLIMFGSERAENEWEAGAPDDRYKASYPRTFYARLNVNNWNADDIEWVNITDQIYQGGIVNSGVGVGSVVVKDNYIYYMFGGEDHF
NPWTYGDNSAKDPFKSDGHPSDLYCYKMKIGPDNRVSRDFRYGAVPNRAVPVFFDTNGVRTVPAPMEFTGDLGLGHVTIRASTSSNIRSEVL
MEGEYGFIGKSIPTDNPAGQRIIFCGGEGTSSTTGAQITLYGANNTDSRRIVYNGDEHLFQSADVKPYNDNVTALGGPSNRFTTAYLGSNPI
VTSNGERKTEPVVFDDAFLDAWGDVHYIMYQWLDAVQLKGNDARIHFGVIAQQIRDVFIAHGLMDENSTNCRYAVLCYDKYPRMTDTVFSHN
EIVEHTDEEGNVTTTEEPVYTEVVIHEEGEEWGVRPDGIFFAEAAYQRRKLERIEARLSALEQK

> Tailspike_R2-K11

MTTNTPKYGGLLTDIGAAALATASAAGKKWQPTHMLIGDAGGAPGDTPDPLPSAAQKSLINQRHRAQLNRLFVSDKNANTLVAEVVLPVEVG
GFWIREIGLQDADGKFVAVSNCPPSYKAAMESGSARTQTIRVNIALSGLENVQLLIDNGIIYATQDWVKEKLGSGVLPLSNLLSTPDGASQI
GTKSGLTVQDYLNGVKSATILRNIEPVIDGQRIVLSEISPTLGPKSGGTLVYDQSDTSSVDDGYTVFVTAGGKRWKREESYIDVAWFGPNFG
LALQTAVNLVDNYVRTVGFYSRKTIYIAAGTYTTDRQVDIPSYVSVVAIGNVSINGSGLPVNSYVLRITNKVGGIVTTHHSGWNLGAVGGTL
RLVGNGNTGQVDGLYVGGATSMSDVRNVSLYAVSTSGVRYGLTFGSTNTYLFTATKCHFETSLVNLYIPGTTSSNSGEKMVFNDTVFGGSSR
NHVEVSTPGMDLTFNNCSFDFTSGSVLYGTETWGYAKVGMNNCHFEGFNSLWIKVDAPQGGFIGSNRAITVSNATVLPRLRSNTAGTNSASR
MHIDAKSTPVYISGLDLRHEVVPYTEEIFMASAETTLSLQGYLKDPHFQIPSAAHIQNRGWNIADETTGTVVNSPATLDSLTRFTCTERNAM
SAAVVDGGTSGKLLAMTGAGGYFTLVTKGFIPVSTFQRIGGAMSIQAAASTGNIQCTLGVQWFDYDGNLIGTDQAFAINMREVFNNSSLPNF
AEGNNRFISTSARTFRAPAGAAKCKPLWRISGHTGVVNISRLASFVL

>Tailspike_R2-K31

MTTNTPKYGGLLTDIGAAALATASAAGKKWQPTHMLIGDAGGAPGDTPDPLPSAAQKSLINQRHRAQLNRLFVSDKNANTLVAEVVLPVEVG
GFWIREIGLQDADGKFVAVSNCPPSYKAAMESGSARTQTIRVNIALSGLENVQLLIDNGIIYATQDWVKEKGSLNDLNQPRGSTLGLFTPNL
PLKKRLDTLPNILDFDSDSLNDDSTRFQKAITAGVKSLYVPEPQFFGNNKPLKIANVDIVTNMHIYGNGSAGYRQVGGAITILDGADYGFKL
AGVDSQTRNIGGRIDGLSFQGEFPTTVADAIRCQSASSFALVNLSFRNLSGSALDLRDFMESHIEHCYFNSVGSDTKNPINIGDFVGSAPWN
VNNLHIENNTFGSCSGNIINISDSANADLIWILNNKFEWDSTPVSPNVSNKAVAYIGRAERVNVSGNGFVYYYPAHNKYDALIRVSDKSAYG
NLFSDNTAWGCTPPSGSDLTPAFYWDIAGGSSAGSNNKANTNLPTRCTSIHSQDIDEPLVRTTPGNRPNLQSIGAMSPGYLSAHSLGGANAS
NFFVPDTGATKYGTVLEAQTGGEVRRLFIPKDIVSQRACVRVQARVMPSPTADALVGLTCDGSIVSTTIQGATQDYHTVAAGGGWQIVEWLI

PASSYTAGQLIFTNRSDTVKFKLDGVRVSRADFVDVTIAWSPTPISAGSVVNTTASITRVSSHVVGTSGLKTDGTLGGAVSSSYFNRGANTL
VVQLAALTAATPSITQVTVRLFLN

>Tailspike_R2-K63-a

MTTNTPKYGGLLTDIGAAALATASAAGKKWQPTHMLIGDAGGAPGDTPDPLPSAAQKSLINQRHRAQLNRLFVSDKNANTLVAEVVLPVEVG
GFWIREIGLQDADGKFVAVSNCPPSYKAAMESGSARTQTIRVNIALSGLENVQLLIDNGIIYATQDWVKEKGSALTKLVDAGAWQVEVAPAG
TVQDSLVFLSPRNFGGVPGTGVDSVAAIEAALAAGDVDLGGEHWFISRPIYCVSGRTIQNGKISTLAAQGSGFMAGSIFAPGNYHPVYVDPV
PKLACSSTNGSATITVSSHEFVVGDLVRLSSTRGIIGSDAVLVPWYMQLARVVGVSGDTVKLDAPIDTTETLVVHKATPAGYNARFNKPLFV
LERATFRNIEVDTWDYWTADSATFECAFEGIRGKARSVVYGNTFCRTNFDNIDITFSNKASEMAFGSHDTNLSNIKFRADSQNWDSTNSVGI
SWAESGRRCTLDNWQLLVPQGVNLSVLVRISSHRDVQIRKGFIQVHSSSNNILSVEHYGGDRPPCNNILFEDIDVNATGAAAVVVDVYKSAN
DSAINAVRFEGISYRGATPSVALMRQRGTTSNQVTGVRASLYSANGGAFLVSSAMAWDVRLYGPGLQVPAAVAVLGRTAVLSASRSNLHALR
WVTEAIIGVTSTTPGNVLREAVIPAGTLRASDYIDFVISGSTGGATSTKDVLVGVLDSGSNFQGVGFTAPTTEESYYTVQGRISFPTTGNCL
ITATIGRAGVGVSYARTLVSISNYTTNDVKLQVQAWVGSSAGSLSVQHGCFAPSELTG

>Tailspike_R2-K63-b

MTTNTPKYGGLLTDIGAAALATASAAGKKWQPTHMLIGDAGGAPGDTPDPLPSAAQKSLINQRHRAQLNRLFVSDKNANTLVAEVVLPVEVG
GFWIREIGLQDADGKFVAVSNCPPSYKAAMESGSARTQTIRVNIALSGLENVQLLIDNGIIYATQDWVKEKGSSAAAAAASENAAKTSENNA
KSSEVAAENARDQVQQIINDAGDASTLVVLANPDGFRHIGRCKDIATLRTIEPVESRQVIEVLSYYNGLAQGGGTFWYDPNDSVTEDNGGSC
IVTNGGKRWKRIIDGAVDVLSFGAKPDDISFDSAPHIQAALDNHDAVSLYGRSYYIGSPIYMPSRTVFDGMGGKLTSIAPSTAGFMAGSIFA
PGNYHPDFWEEVPKVAATTTLGSANITLADPNIVNVGDIIRLSSTTGVLSAGFFVSEYLQMARVLSKTGNVITIDGPVESQLTLVAANANQP
GYLARFNKPLFCCVDSIIQNIEIDTWDYWTADSATYNVKFSNIWGSAKAVAYGNTFCRSLFEDIRIVFSGRVSELAFGSHDTNLVRITAIAS
PKGLSASVVFGWAESGRRCTIDTFSIMLNANANPSTVIRVSGHRDSLIKNGSIYVHNNTNNILSVENYGTTADGVRPDCDNITFENVSIFVT
GSSAVVCDVYKSADNSVIKNVAFKNIKYFGPTPSVALYRARGTLANFVKGVQANISSDTGGAIVLSNSENNVLTFTGPVSVTSLVSAAAKNT
LSIRNYARSSAKANNFTQESTLNVTDTTANAVSKEFTYPAGSLRINDKIKLSLGGSTAGTVGKKTVQVGFIGSDGAFKYVELAALATDQVYW
TMEVEISFLRTTNSQTNELETSAIITSFLSKGAATGAALSGSRALAVVSDLALSNFVVQVRAWKENAADGLSLSRMNLQLEDLTA

> Tailspike_R2-K11-K1_K11

MTTNTPKYGGLLTDIGAAALATASAAGKKWQPTHMLIGDAGGAPGDTPDPLPSAAQKSLINQRHRAQLNRLFVSDKNANTLVAEVVLPVEVG
GFWIREIGLQDADGKFVAVSNCPPSYKAAMESGSARTQTIRVNIALSGLENVQLLIDNGIIYATQDWVKEKGSADDTTSLKGINWVYNNGSA
NGGETSILITREGPVFAVPTIYINGDRQSVGYHYSYDSGDKTIHLVKPLTAGDFVECVTSEGVLPLSNLLSTPDGASQIGTKSGLTVQDYLN
GVKSATILRNIEPVIDGQRIVLSEISPTLGPKSGGTLVYDQSDTSSVDDGYTVFVTAGGKRWKREESYIDVAWFGPNFGLALQTAVNLVDNY
VRTVGFYSRKTIYIAAGTYTTDRQVDIPSYVSVVAIGNVSINGSGLPVNSYVLRITNKVGGIVTTHHSGWNLGAVGGTLRLVGNGNTGQVDG
LYVGGATSMSDVRNVSLYAVSTSGVRYGLTFGSTNTYLFTATKCHFETSLVNLYIPGTTSSNSGEKMVFNDTVFGGSSRNHVEVSTPGMDLT
FNNCSFDFTSGSVLYGTETWGYAKVGMNNCHFEGFNSLWIKVDAPQGGFIGSNRAITVSNATVLPRLRSNTAGTNSASRMHIDAKSTPVYIS
GLDLRHEVVPYTEEIFMASAETTLSLQGYLKDPHFQIPSAAHIQNRGWNIADETTGTVVNSPATLDSLTRFTCTERNAMSAAVVDGGTSGKL
LAMTGAGGYFTLVTKGFIPVSTFQRIGGAMSIQAAASTGNIQCTLGVQWFDYDGNLIGTDQAFAINMREVFNNSSLPNFAEGNNRFISTSAR
TFRAPAGAAKCKPLWRISGHTGVVNISRLASFVL

>Tailspike_R2-K11-K1_K1

MLDNFNQPKGSTIGVLKDGRTIQEAFDSLPRLESFSGAKGDGVTDDTAALTSALNDTPVGQKINGNGKTYKVTSLPDISRFINTRFVYERIP
GQPLYYASEEFVQGELFKITDTPYYNAWPQDKAFVYENVIYAPYMGSDRHGVSRLHVSWVKSGDDGQTWSTPEWLTDLHPDYPTVNYHCMSM
GVCRNRLFAMIETRTLAKNALTNCALWDRPMSRSLHLTGGITKAANQRYATIHVPDHGLFVGDFVNFSNSAVTGVSGDMTVATVIDKDNFTV
LTPNQQTSDLNNAGKNWHMGTSFHKSPWRKTDLGLIPSVTEVHSFATIDNNGFAMGYHQGDVAPREVGLFYFPDAFNSPSNYVRRQIPSEYE
PDASEPCIKYYDGVLYLITRGTRGDRLGSSLHRSRDIGQTWESLRFPHNVHHTTLPFAKVGDDLIMFGSERAENEWEAGAPDDRYKASYPRT
FYARLNVNNWNADDIEWVNITDQIYQGGIVNSGVGVGSVVVKDNYIYYMFGGEDHFNPWTYGDNSAKDPFKSDGHPSDLYCYKMKIGPDNRV
SRDFRYGAVPNRAVPVFFDTNGVRTVPAPMEFTGDLGLGHVTIRASTSSNIRSEVLMEGEYGFIGKSIPTDNPAGQRIIFCGGEGTSSTTGA
QITLYGANNTDSRRIVYNGDEHLFQSADVKPYNDNVTALGGPSNRFTTAYLGSNPIVTSNGERKTEPVVFDDAFLDAWGDVHYIMYQWLDAV
QLKGNDARIHFGVIAQQIRDVFIAHGLMDENSTNCRYAVLCYDKYPRMTDTVFSHNEIVEHTDEEGNVTTTEEPVYTEVVIHEEGEEWGVRP
DGIFFAEAAYQRRKLERIEARLSALEQK

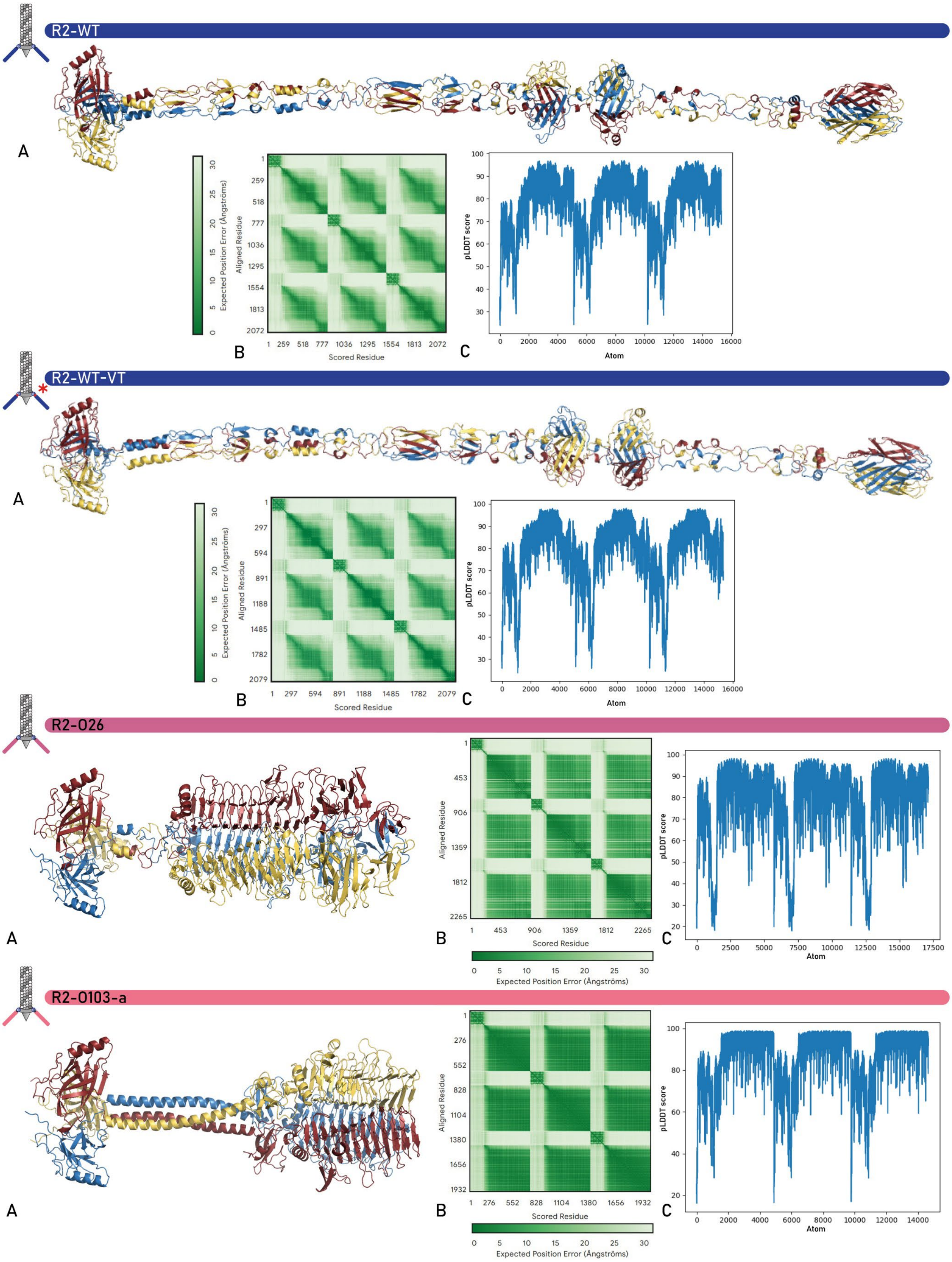
R2-WT
A
Expected Position Error (Ångströms)
Aligned Residue
Scored Residue
B
pLDDT score
Atom
C
R2-WT-VT
A
Expected Position Error (Ångströms)
Aligned Residue
Scored Residue
B
pLDDT score
Atom
C
R2-026
A
Aligned Residue
Scored Residue
Expected Position Error (Ångströms)
B
pLDDT score
Atom
C
R2-0103-a
A
Aligned Residue
Scored Residue
Expected Position Error (Ångströms)
B
pLDDT score
Atom
C

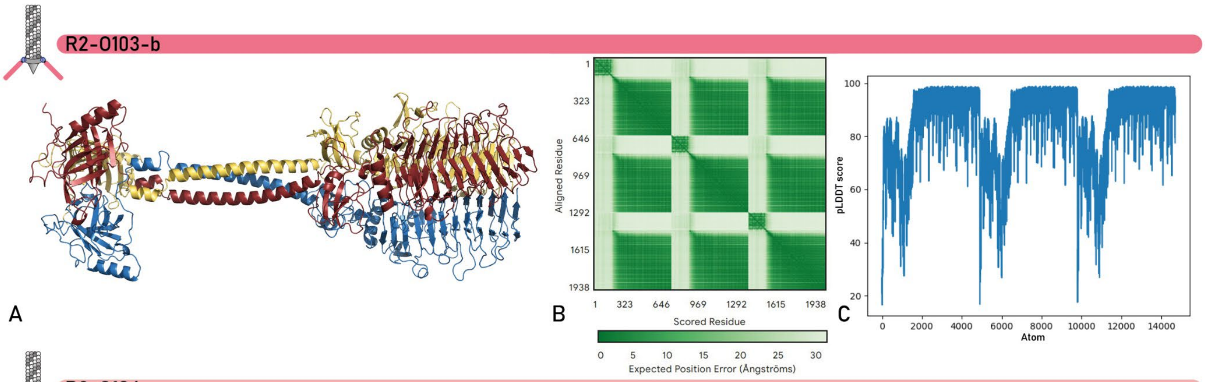
R2-0103-b
A
B
C
Aligned Residue
1 323 646 969 1292 1615 1938
Scored Residue
0 5 10 15 20 25 30
Expected Position Error (Ångströms)
pLDDT score
20 40 60 80 100
0 2000 4000 6000 8000 10000 12000 14000
Atom

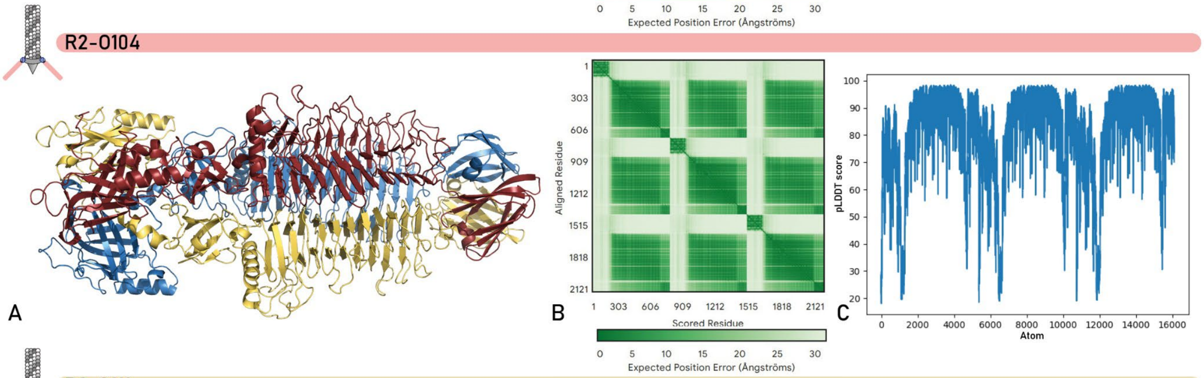
R2-0104
A
B
C
Aligned Residue
1 303 606 909 1212 1515 1818 2121
Scored Residue
0 5 10 15 20 25 30
Expected Position Error (Ångströms)
pLDDT score
20 30 40 50 60 70 80 90 100
0 2000 4000 6000 8000 10000 12000 14000 16000
Atom

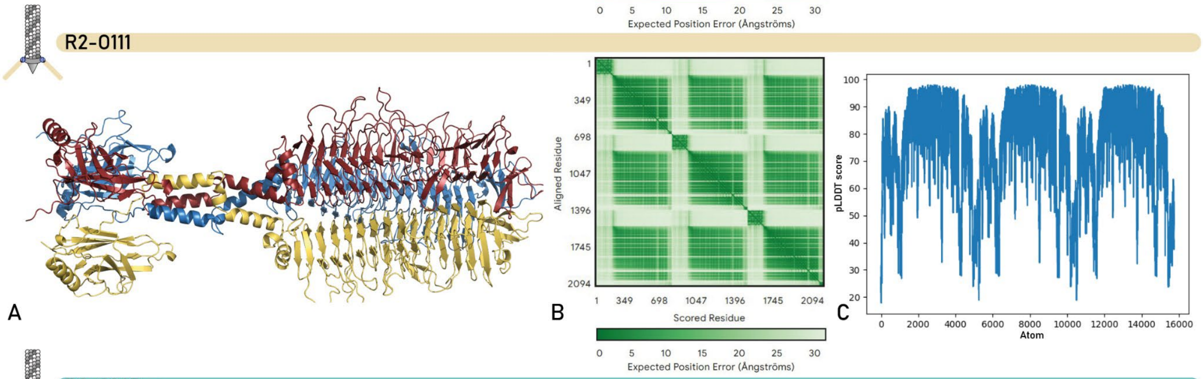
R2-0111
A
B
C
Aligned Residue
1 349 698 1047 1396 1745 2094
Scored Residue
0 5 10 15 20 25 30
Expected Position Error (Ångströms)
pLDDT score
20 30 40 50 60 70 80 90 100
0 2000 4000 6000 8000 10000 12000 14000 16000
Atom

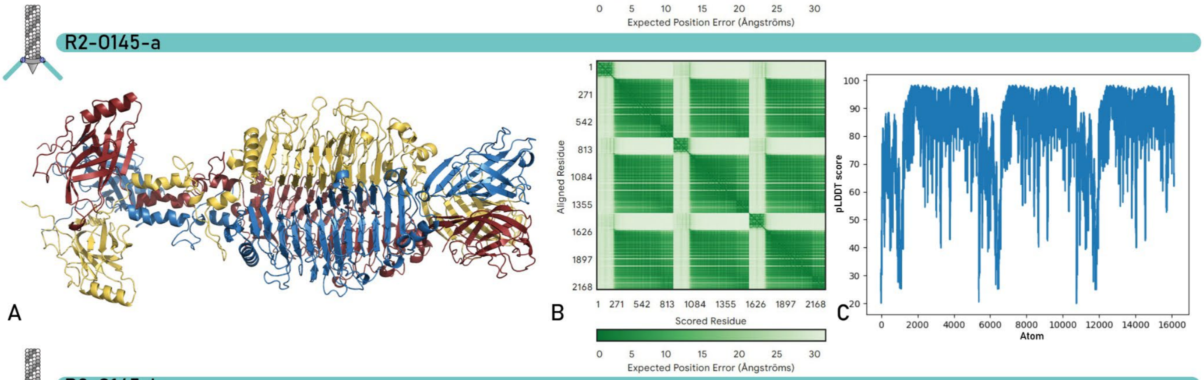
R2-0145-a
A
B
C
Aligned Residue
1 271 542 813 1084 1355 1626 1897 2168
Scored Residue
0 5 10 15 20 25 30
Expected Position Error (Ångströms)
pLDDT score
20 30 40 50 60 70 80 90 100
0 2000 4000 6000 8000 10000 12000 14000 16000
Atom

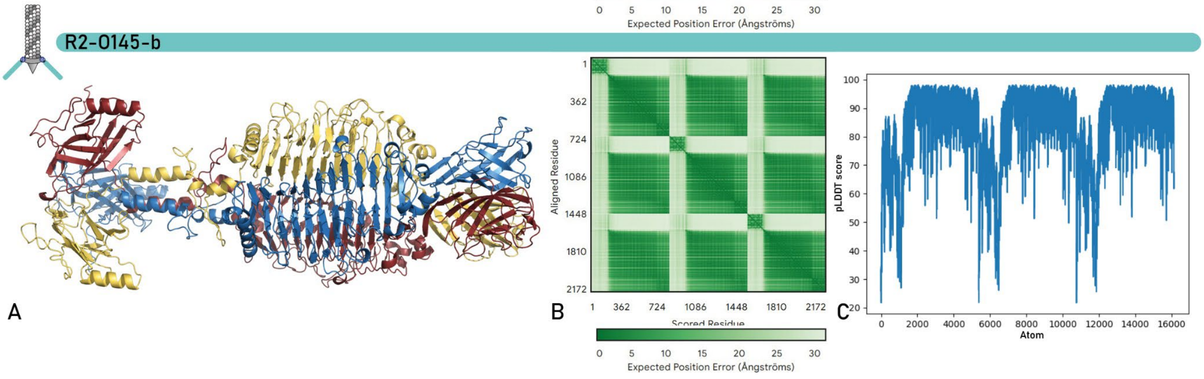
R2-0145-b
A
B
C
Aligned Residue
1 362 724 1086 1448 1810 2172
Scored Residue
0 5 10 15 20 25 30
Expected Position Error (Ångströms)
pLDDT score
20 30 40 50 60 70 80 90 100
0 2000 4000 6000 8000 10000 12000 14000 16000
Atom

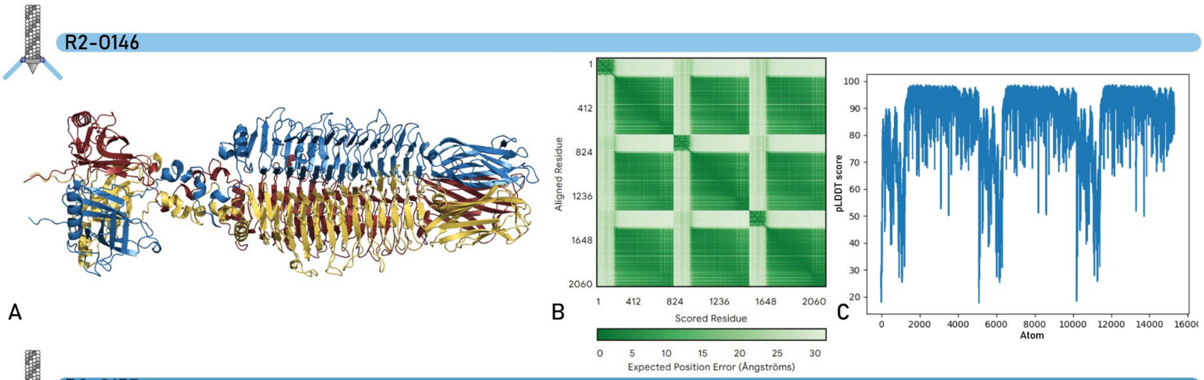
R2-0146
A
B
C
Aligned Residue
Scored Residue
Expected Position Error (Ångströms)
pLDDT score
Atom

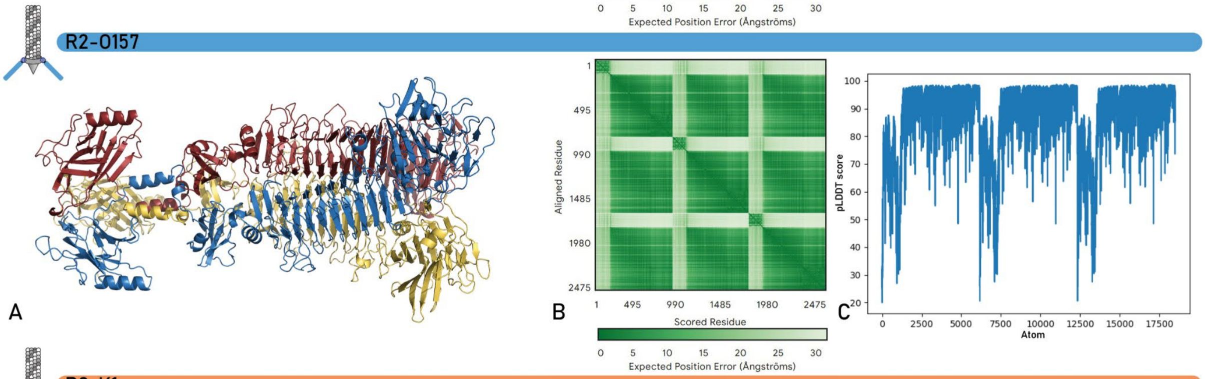
R2-0157
A
B
C
Aligned Residue
Scored Residue
Expected Position Error (Ångströms)
pLDDT score
Atom

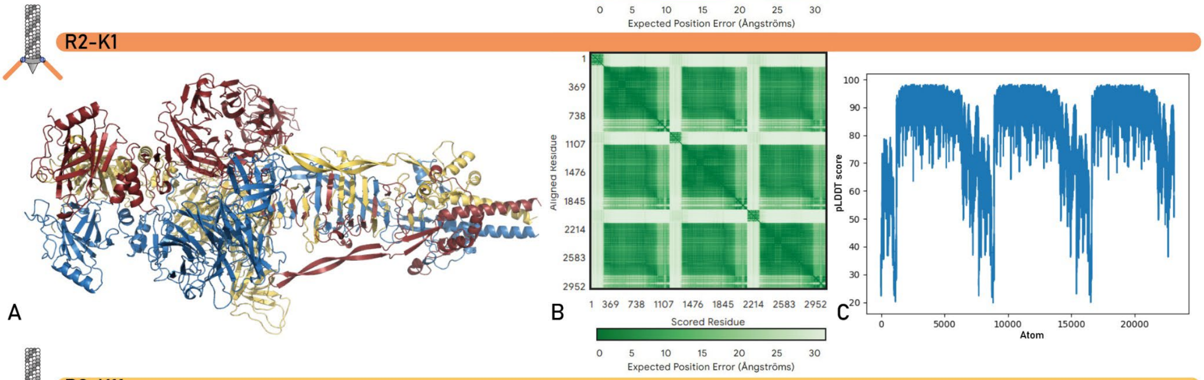
R2-K1
A
B
C
Aligned Residue
Scored Residue
Expected Position Error (Ångströms)
pLDDT score
Atom

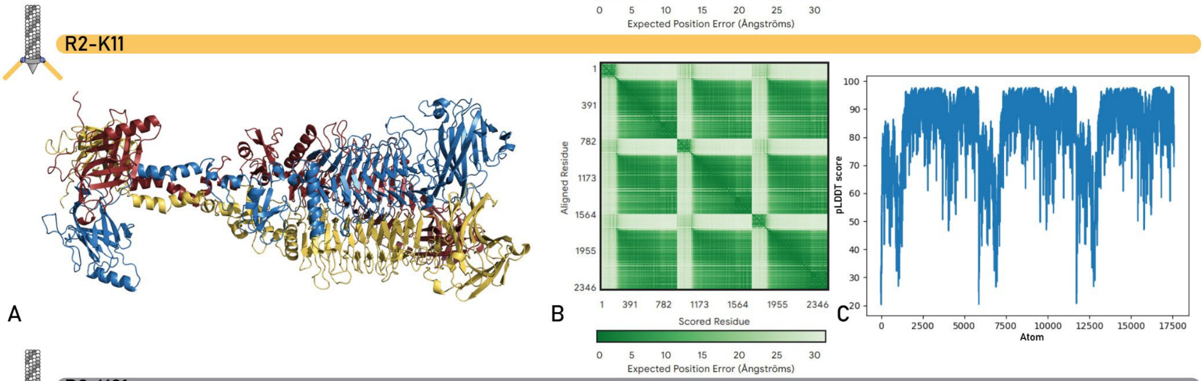
R2-K11
A
B
C
Aligned Residue
Scored Residue
Expected Position Error (Ångströms)
pLDDT score
Atom

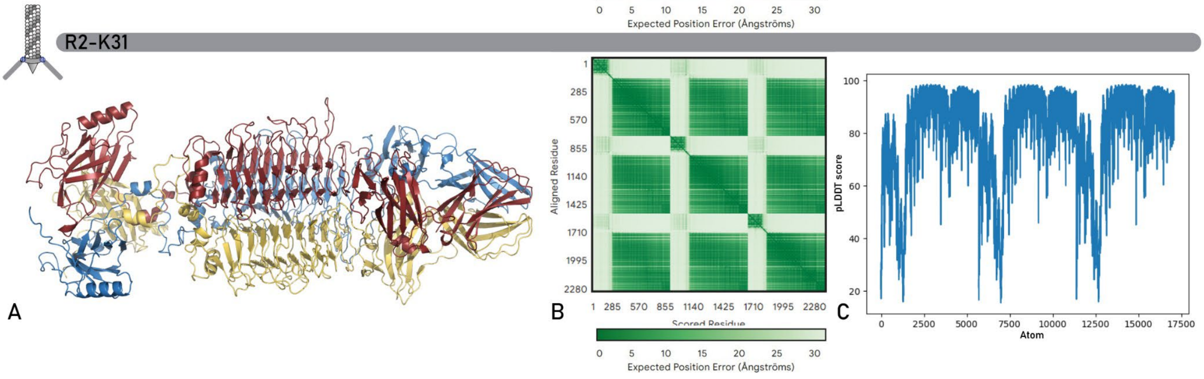
R2-K31
A
B
C
Aligned Residue
Expected Position Error (Ångströms)
pLDDT score
Atom

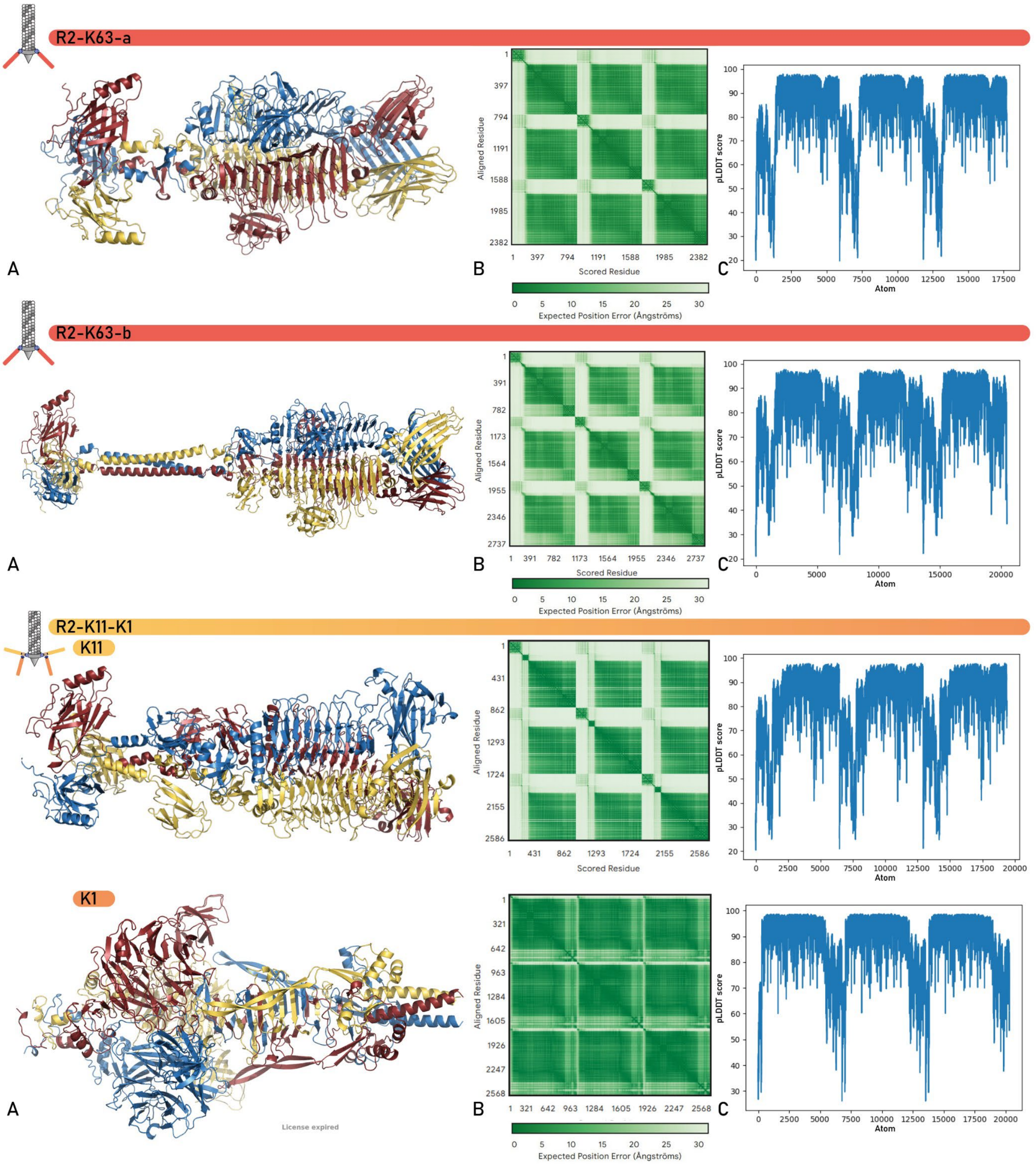

Supplementary Figure S1. AlphaFold3 predicted receptor-binding protein (RBP) structures. Each chimeric RBP is shown according to their target O-antigen/capsule serotype. (**A**) AlphaFold3 structure of trimeric RBP with each trimer indicated in a different color. (**B**) AlphaFold3 expected error per amino acid residue of the RBP. (**C**) Predicted Local Distance Difference Test (pLDDT) scores per atom within the RBP.

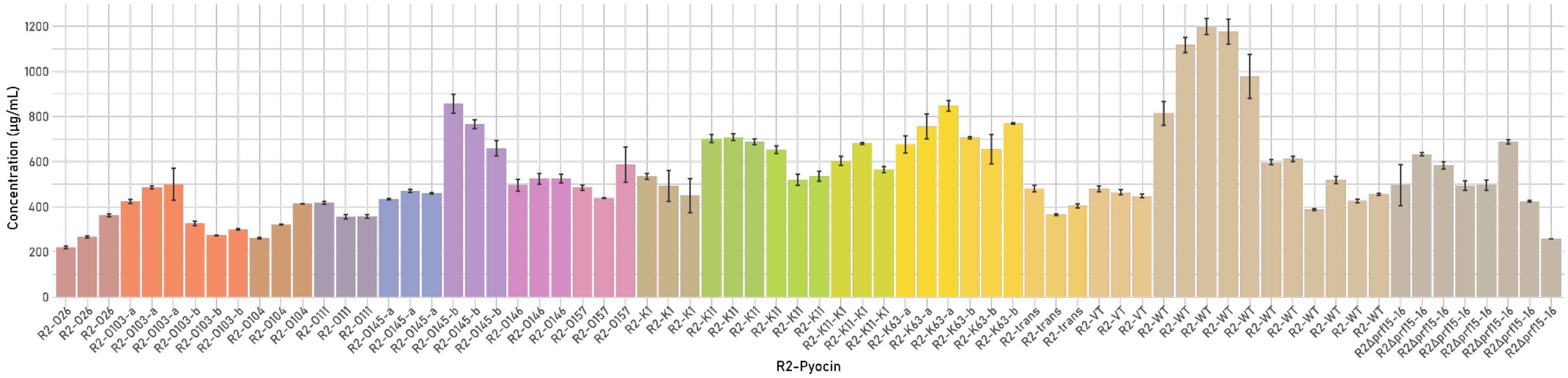

Supplementary Figure S2. An overview of the protein concentrations for each biological replicate of produced engineered R2 tailocin. Protein concentrations of the expressed (engineered) R2 tailocins were measured with the Micro BCA^{TM} Protein Assay Kit (Thermo Fisher Scientific) after purification with ammonium sulfate (AS) precipitation or high-speed centrifugation. The error bars indicate the standard deviation of technical triplicate measurements.

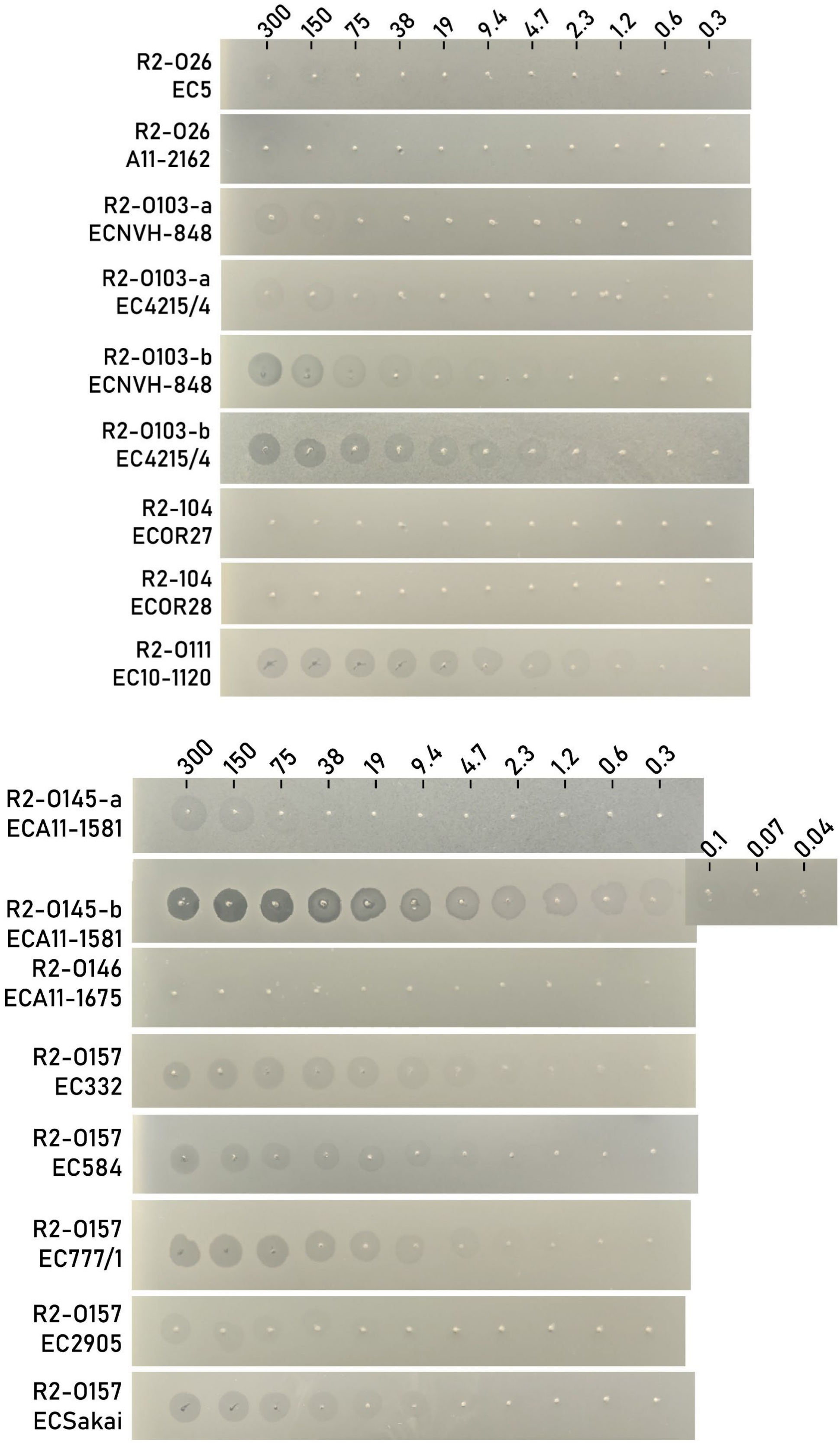
300
150
75
38
19
9.4
4.7
2.3
1.2
0.6
0.3
R2-O26 EC5
R2-O26 A11-2162
R2-O103-a ECNVH-848
R2-O103-a EC4215/4
R2-O103-b ECNVH-848
R2-O103-b EC4215/4
R2-104 ECOR27
R2-104 ECOR28
R2-O111 EC10-1120
300
150
75
38
19
9.4
4.7
2.3
1.2
0.6
0.3
0.1
0.07
0.04
R2-O145-a ECA11-1581
R2-O145-b ECA11-1581
R2-O146 ECA11-1675
R2-O157 EC332
R2-O157 EC584
R2-O157 EC777/1
R2-O157 EC2905
R2-O157 ECSakai

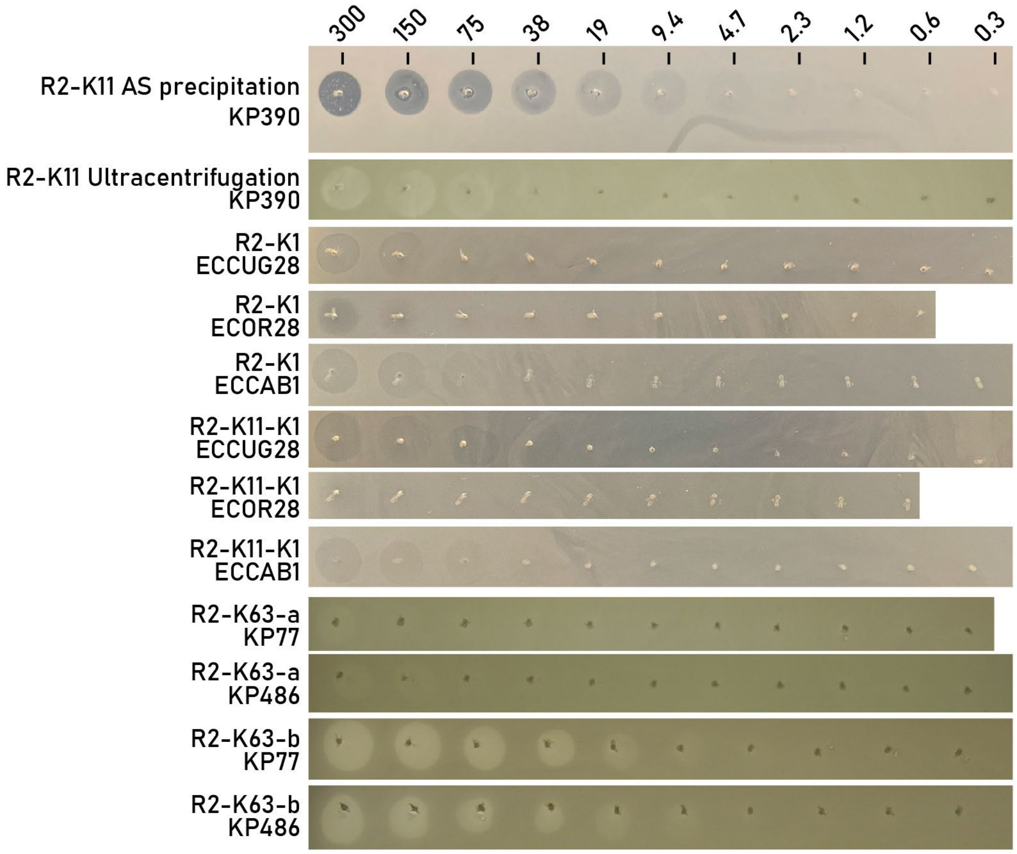

Supplementary Figure S3. Results of the two-fold serial dilution spotting assays of engineered R2 tailocins. The name of the engineered R2 tailocin construct and strain used to perform the spotting are stated on the left side of the figures. Volumes of 5 µL were spotted in increasing dilutions, from 300 to 0.04 µg/µL.

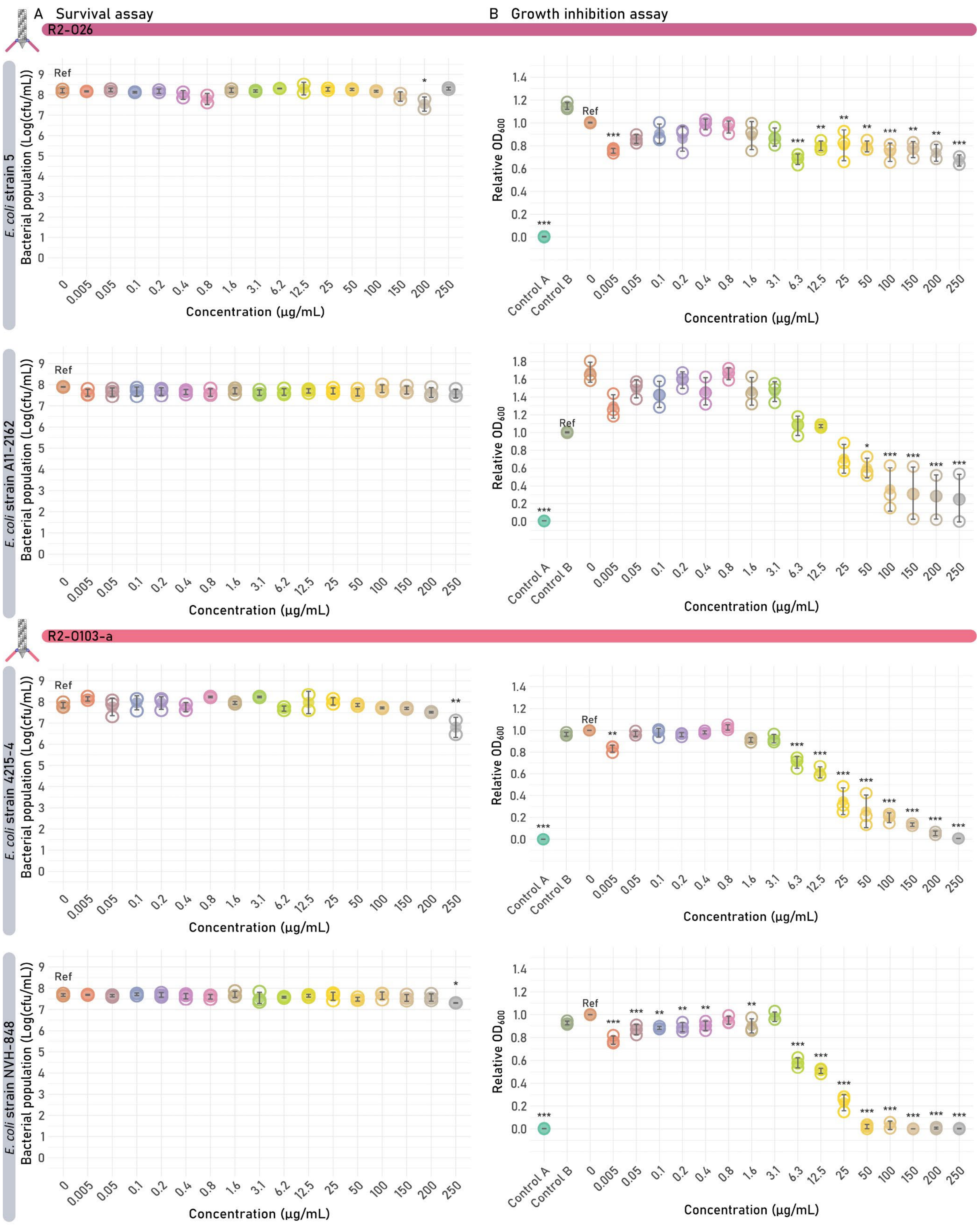
A Survival assay
B Growth inhibition assay
R2-026
R2-0103-a
E. coli strain 5
E. coli strain A11-2162
E. coli strain 4215-4
E. coli strain NVH-848
Bacterial population (Log(cfu/mL))
Relative OD_{600}
Ref
Control A
Control B
Concentration (µg/mL)

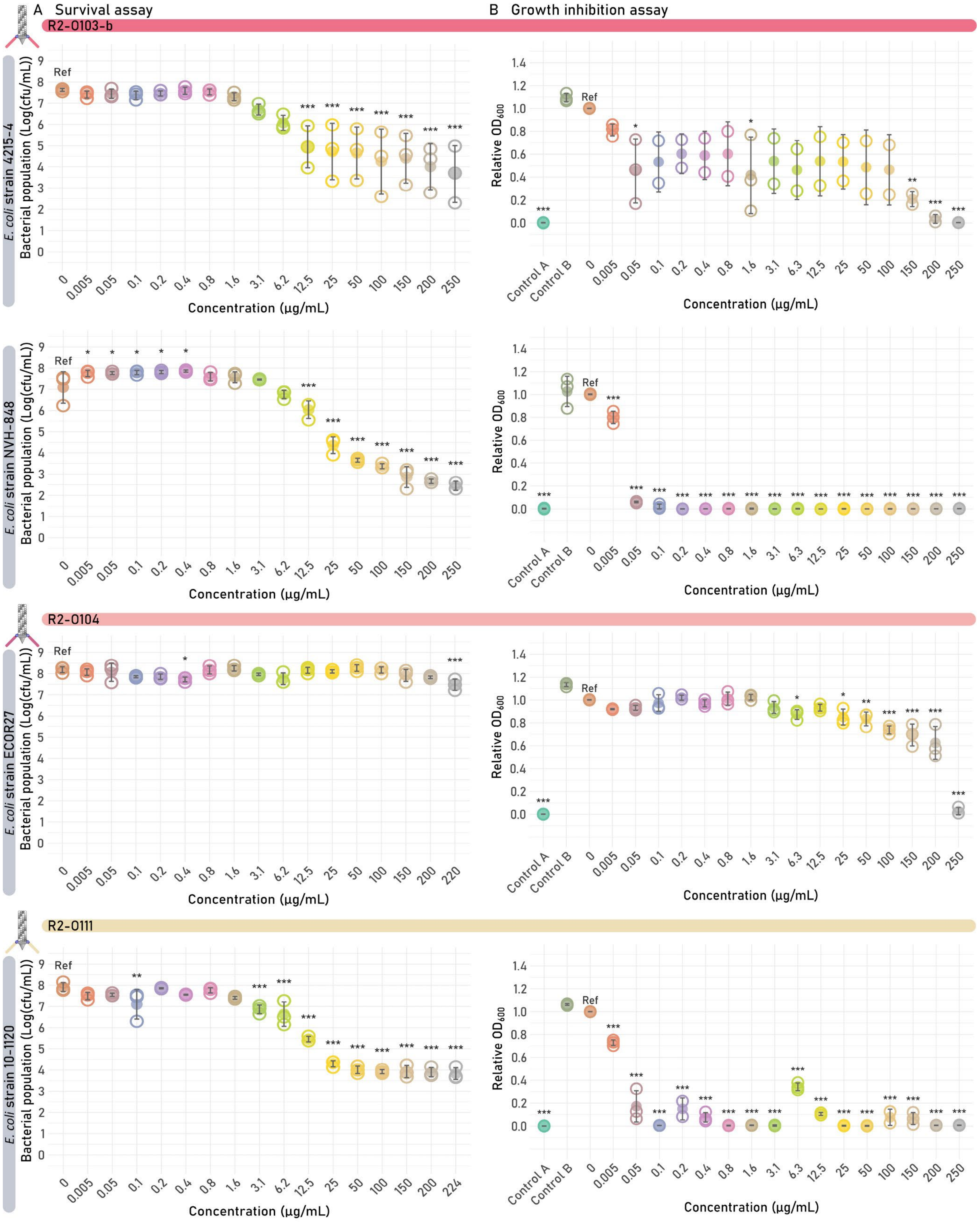

A Survival assay
B Growth inhibition assay
R2-0103-b
E. coli strain 4215-4
E. coli strain NVH-848
R2-0104
E. coli strain ECOR27
R2-0111
E. coli strain 10-1120
Bacterial population (Log(cfu/mL))
Relative OD600
Concentration (µg/mL)
Ref
Control A
Control B

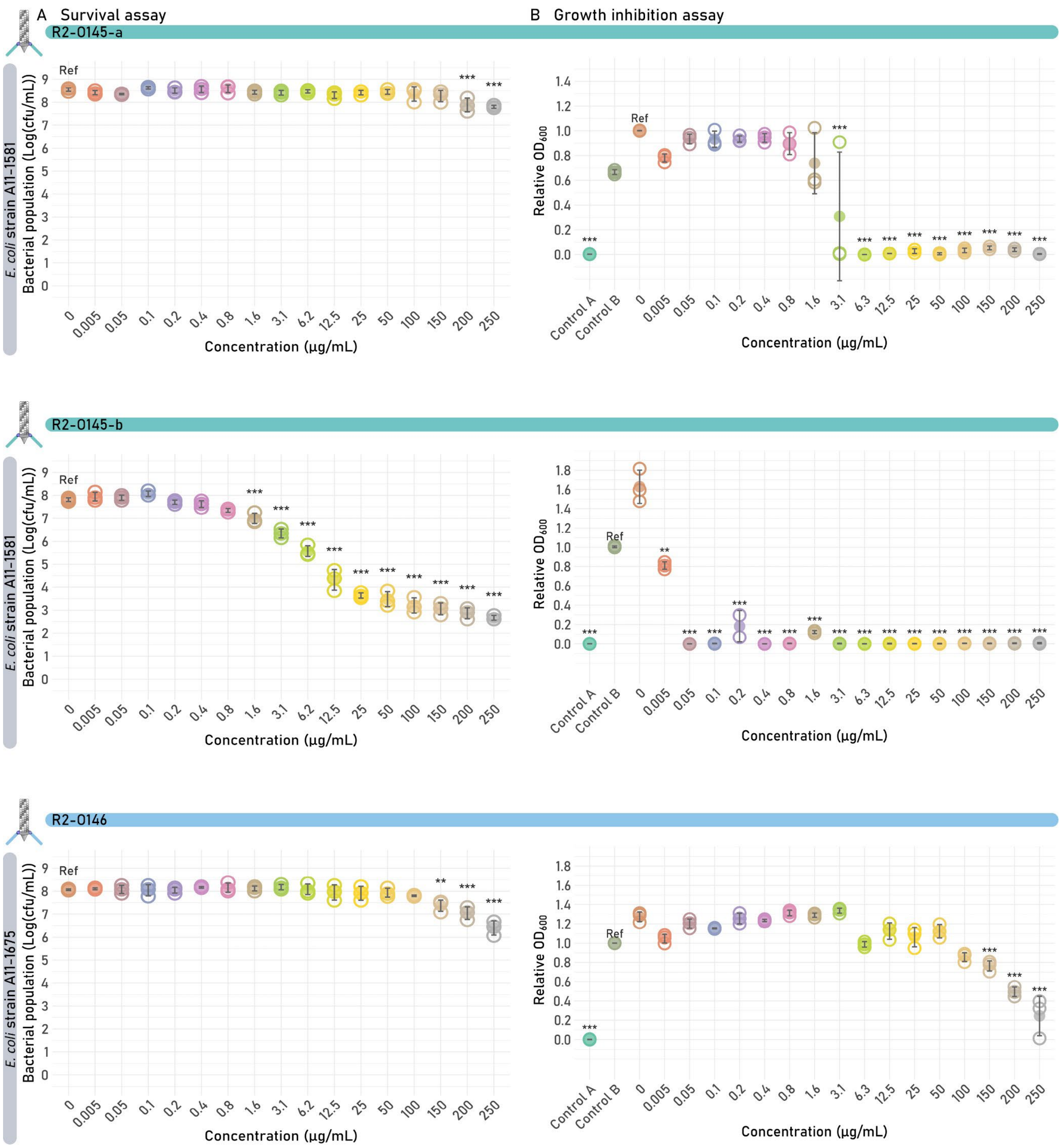
A Survival assay
B Growth inhibition assay
R2-0145-a
R2-0145-b
R2-0146
E. coli strain A11-1581
E. coli strain A11-1675
Bacterial population (Log(cfu/mL))
Relative OD600
Concentration (µg/mL)
Ref
Control A
Control B

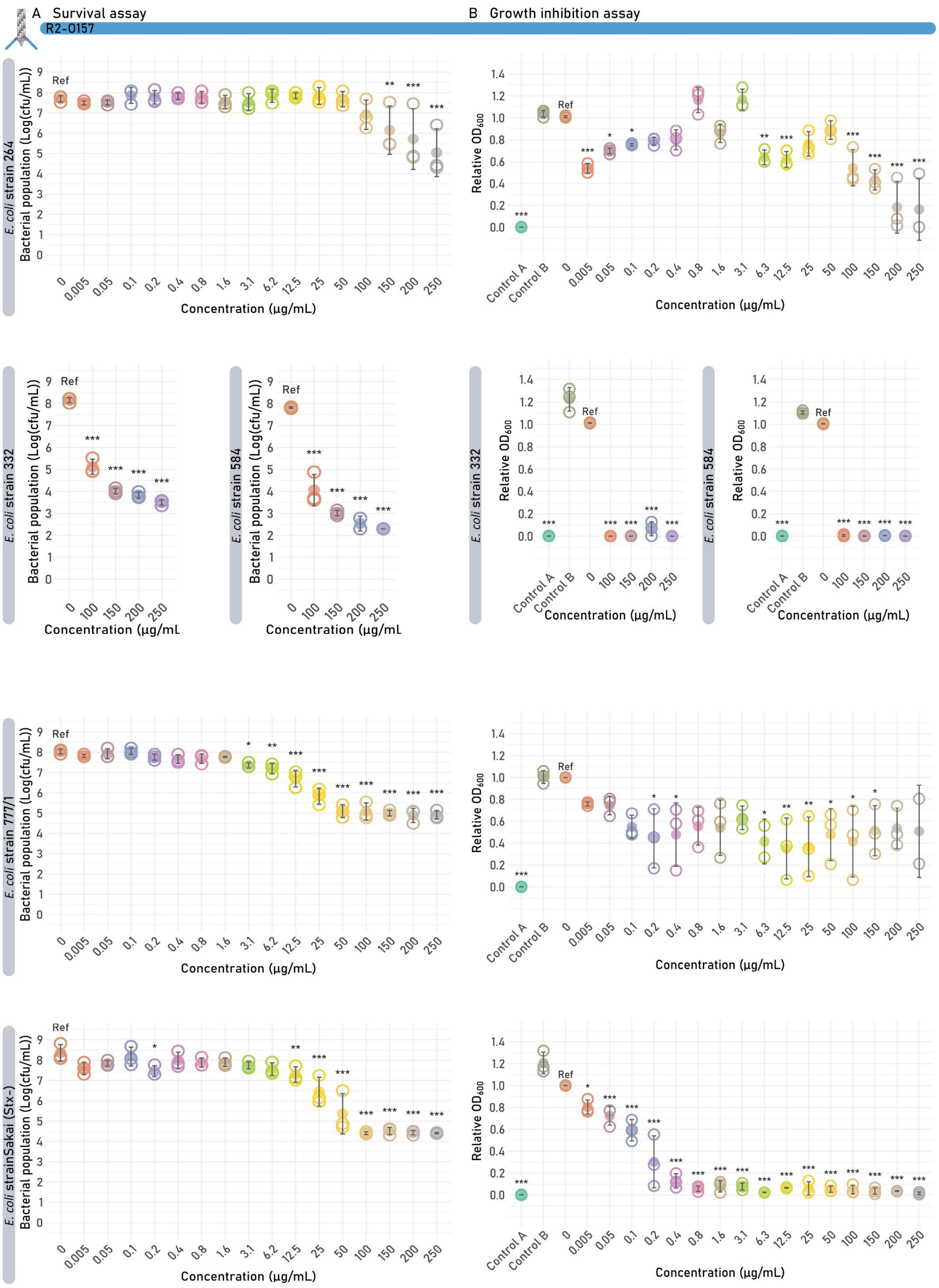
A Survival assay
B Growth inhibition assay
R2-0157
E. coli strain 264
E. coli strain 332
E. coli strain 584
E. coli strain 777/1
E. coli strainSakai (Stx-)
Bacterial population (Log(cfu/mL))
Relative OD600
Concentration (µg/mL)
Ref
Control A
Control B

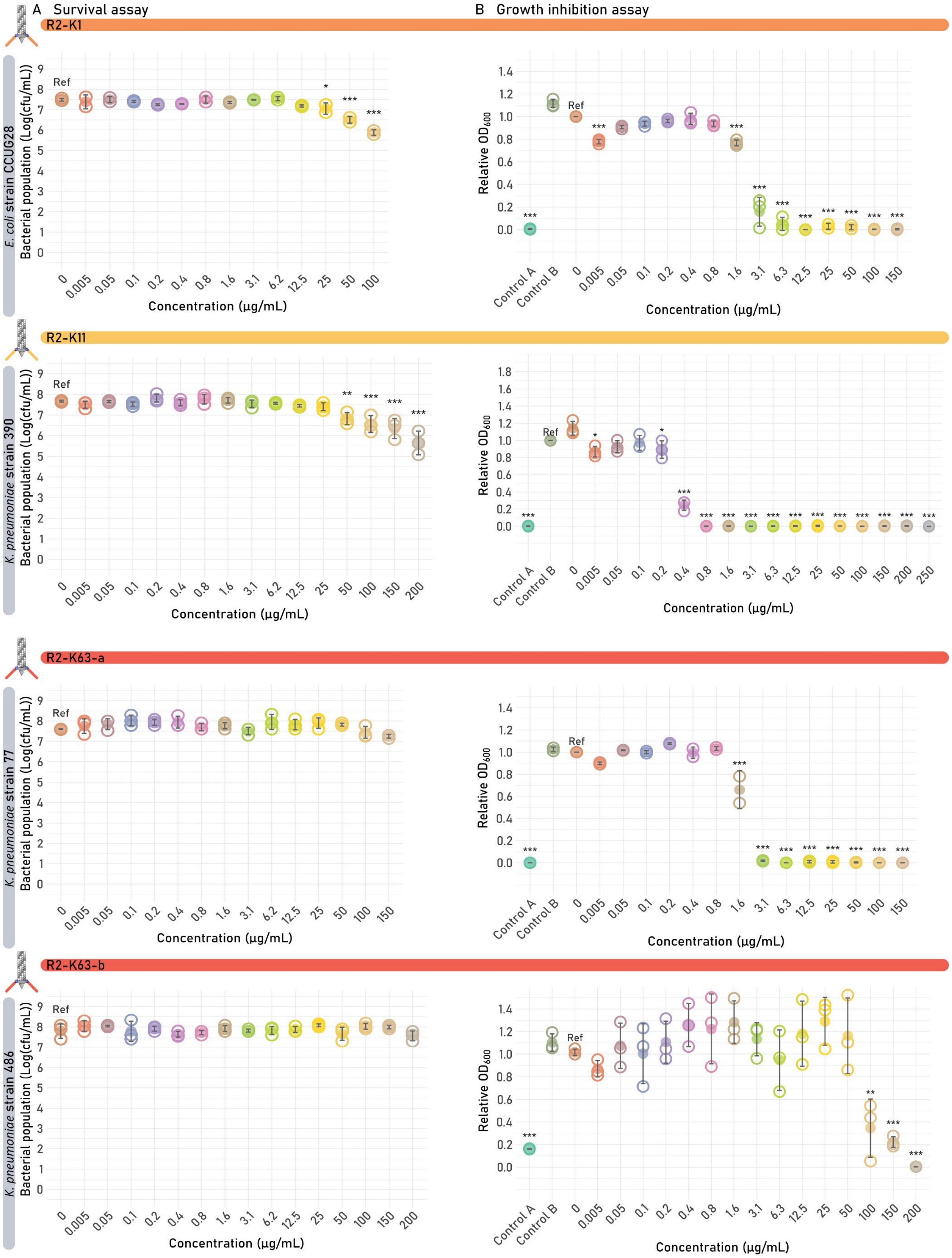
A Survival assay
B Growth inhibition assay
R2-K1
E. coli strain CCUG28
Bacterial population (Log(cfu/mL))
Ref
Concentration (µg/mL)
Relative OD600
Control A
Control B
R2-K11
K. pneumoniae strain 390
R2-K63-a
K. pneumoniae strain 77
R2-K63-b
K. pneumoniae strain 486

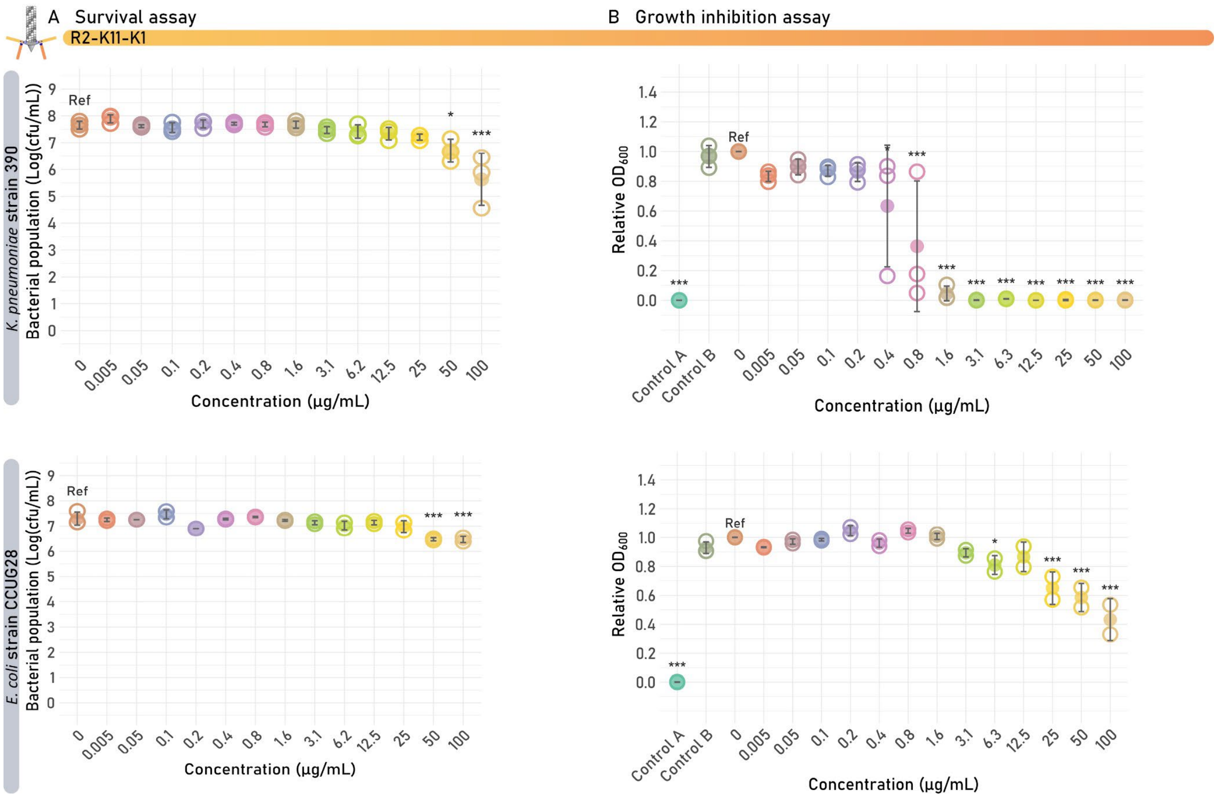

Supplementary Figure S4. Survival and growth inhibition assays of the different engineered R2 tailocins. Each R2 tailocin was tested on their susceptible target strains. Significant differences are shown by asterisks (*$p < 0.05$, **$p < 0.005$, ***$p < 0.001$). Reference (ref) indicates the value that was used as a reference for statistical comparison (untreated sample or control B). (**A**) Results of the survival assay per R2 tailocin construct and tested target strain, showing the bacterial colony count in function of the concentration of the added R2 tailocin. The value of each biological replicate is displayed using open circles and the mean values are shown as filled circles. (B) Growth inhibition assay results at 8 h are shown per R2 tailocin construct and tested target strain. The relative OD_{600} of each biological replicate is displayed using open circles, and the mean relative OD_{600} is shown as full circles. Two additional controls were performed, one containing the R2 tailocin but without the bacterial strain (Control A) and one containing a receptor-binding protein (RBP)-lacking mutant R2 tailocin particle (R2Δ*prf15*) (Control B). Both controls were added at the highest available R2 tailocin concentration (220-250 µg/mL).

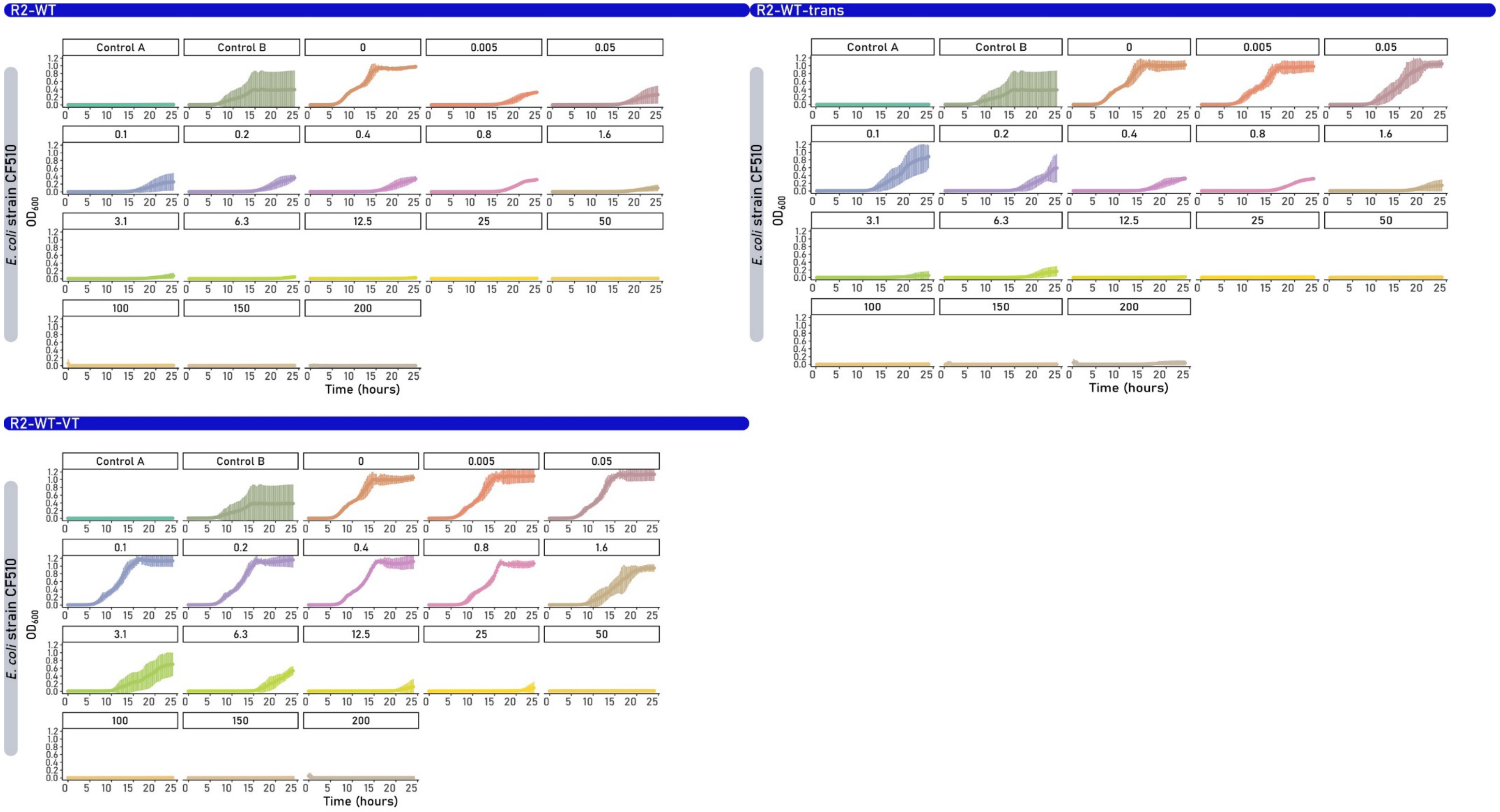
R2-WT
R2-WT-trans
R2-WT-VT
E. coli strain CF510
OD600
Control A
Control B
0
0.005
0.05
0.1
0.2
0.4
0.8
1.6
3.1
6.3
12.5
25
50
100
150
200
Time (hours)

R2-026

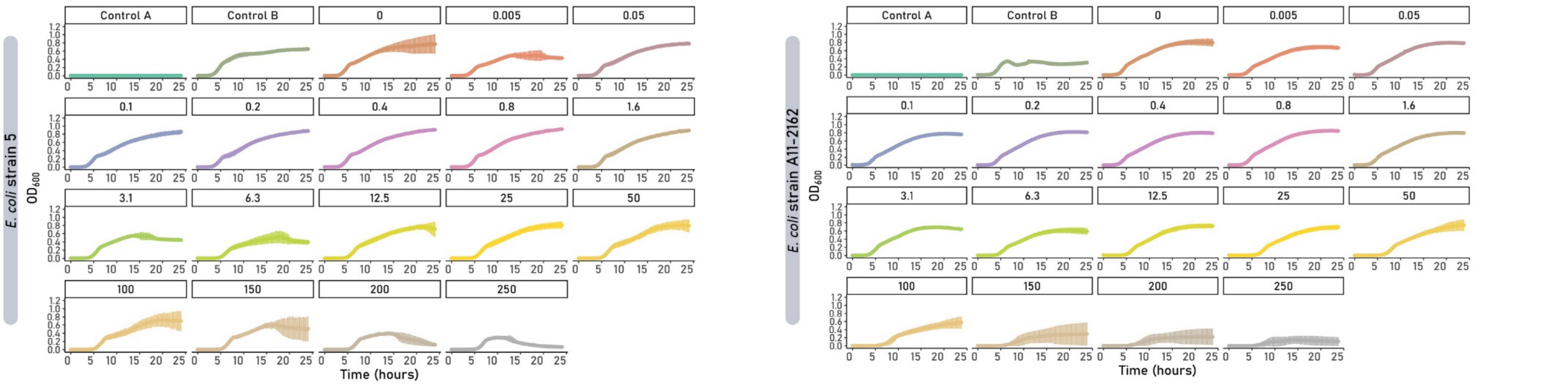

R2-0103-a

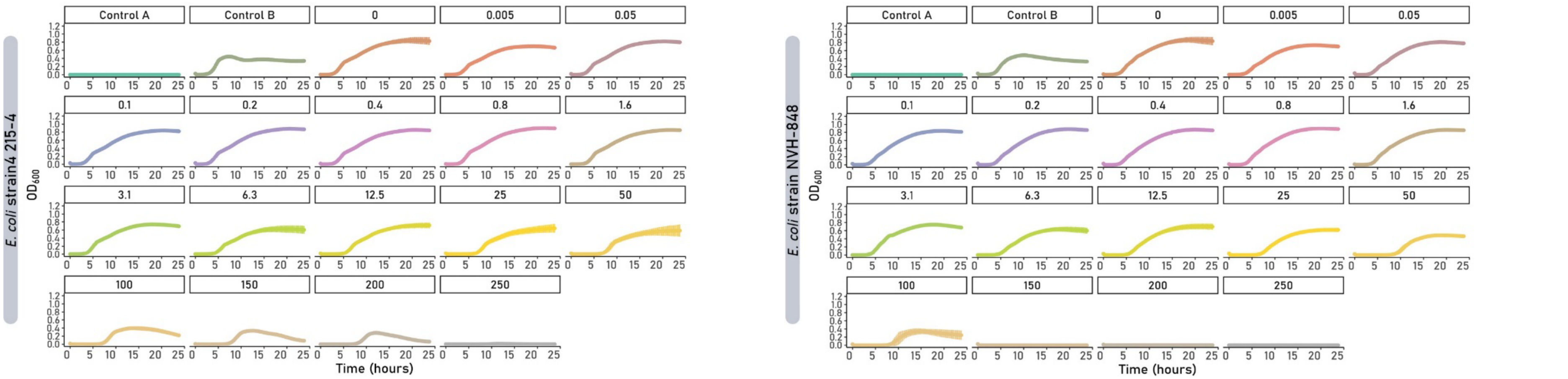

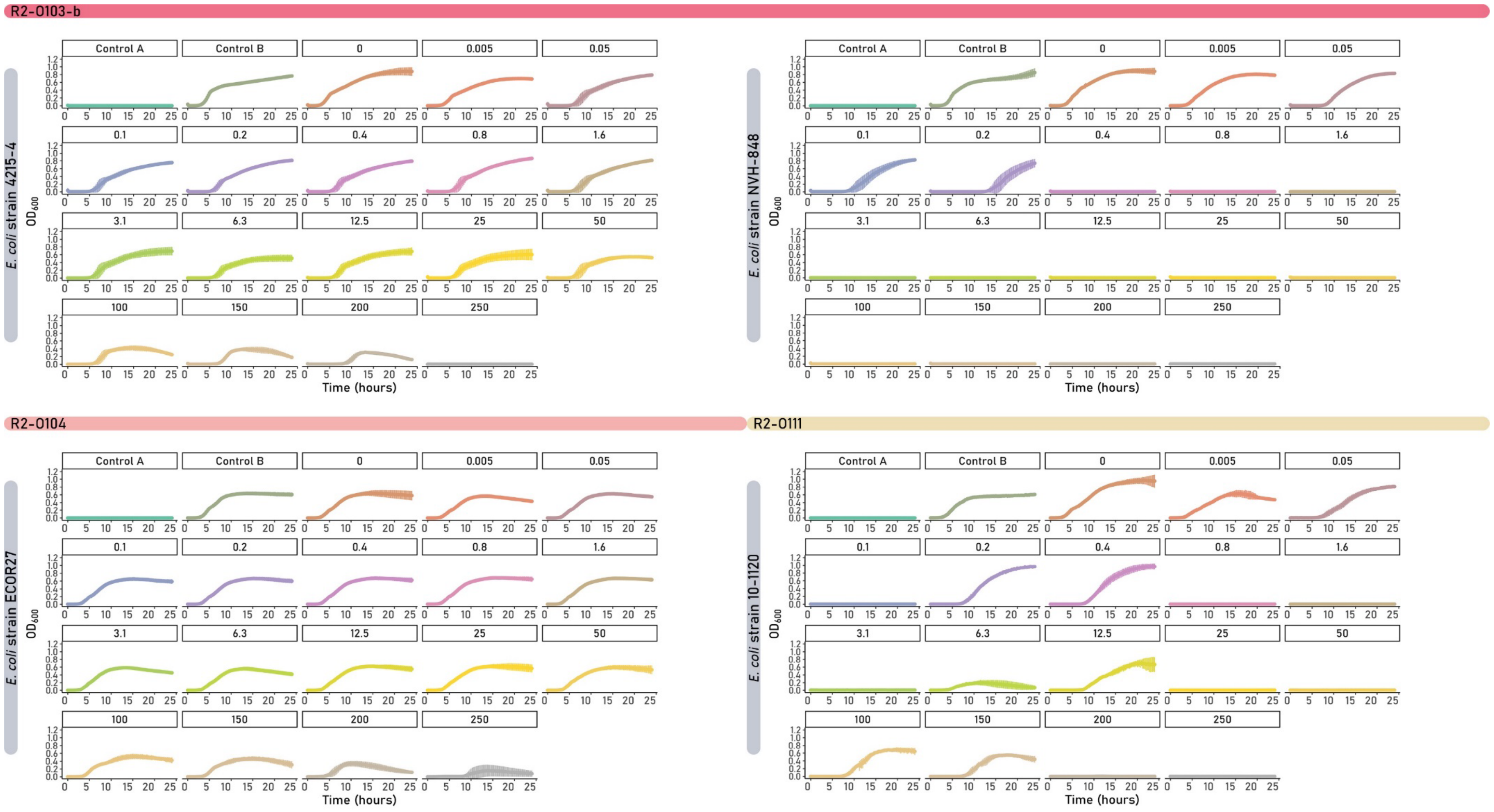

R2-0103-b
E. coli strain 4215-4
E. coli strain NVH-848
R2-0104
E. coli strain ECOR27
R2-0111
E. coli strain 10-1120
Control A
Control B
0
0.005
0.05
0.1
0.2
0.4
0.8
1.6
3.1
6.3
12.5
25
50
100
150
200
250
OD600
Time (hours)

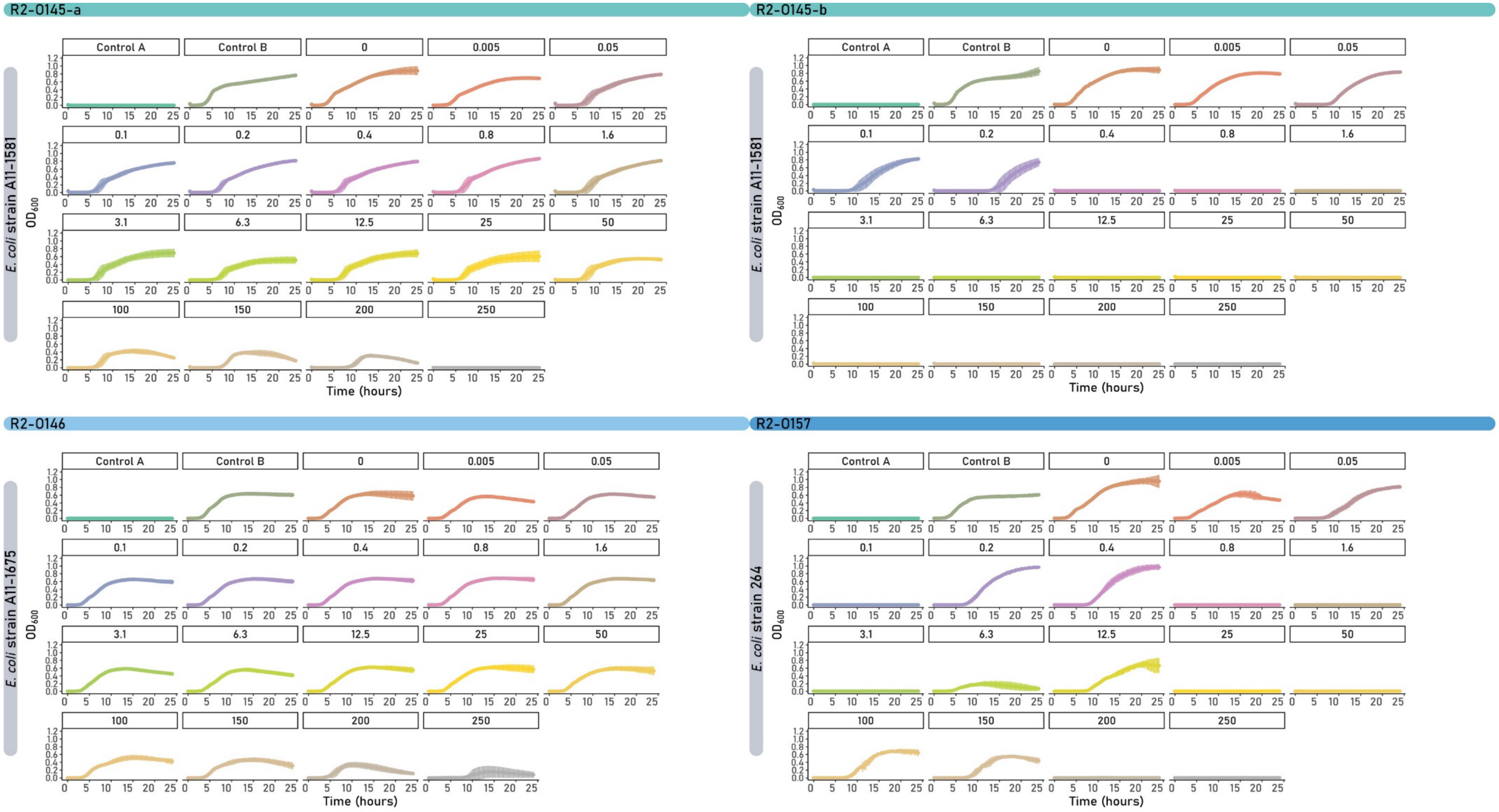

R2-0145-a
E. coli strain A11-1581
OD600
Control A
Control B
0
0.005
0.05
0.1
0.2
0.4
0.8
1.6
3.1
6.3
12.5
25
50
100
150
200
250
Time (hours)
R2-0145-b
E. coli strain A11-1581
OD600
Time (hours)
R2-0146
E. coli strain A11-1675
OD600
Time (hours)
R2-0157
E. coli strain 264
OD600
Time (hours)

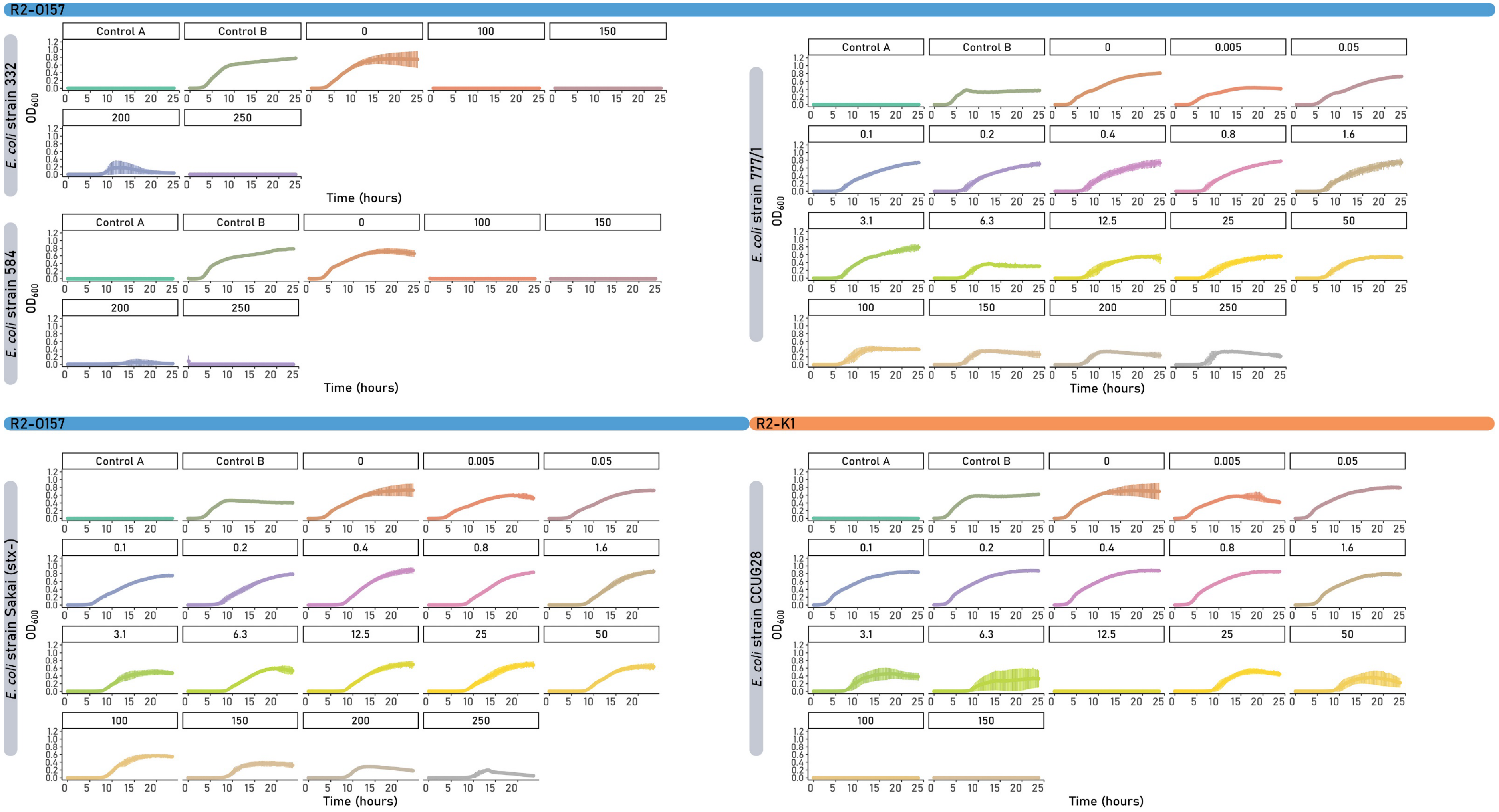

R2-O157
E. coli strain 332
Control A
Control B
0
100
150
200
250
OD600
Time (hours)
E. coli strain 584
Control A
Control B
0
100
150
200
250
OD600
Time (hours)
E. coli strain 777/1
Control A
Control B
0
0.005
0.05
0.1
0.2
0.4
0.8
1.6
3.1
6.3
12.5
25
50
100
150
200
250
OD600
Time (hours)
R2-O157
E. coli strain Sakai (stx-)
Control A
Control B
0
0.005
0.05
0.1
0.2
0.4
0.8
1.6
3.1
6.3
12.5
25
50
100
150
200
250
OD600
Time (hours)
R2-K1
E. coli strain CCUG28
Control A
Control B
0
0.005
0.05
0.1
0.2
0.4
0.8
1.6
3.1
6.3
12.5
25
50
100
150
OD600
Time (hours)

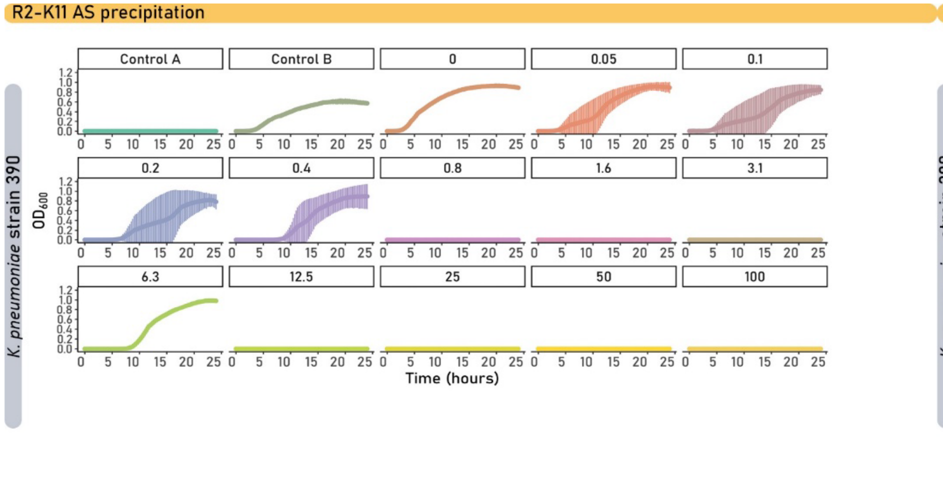
R2-K11 AS precipitation
K. pneumoniae strain 390
OD600
Control A
Control B
0
0.05
0.1
0.2
0.4
0.8
1.6
3.1
6.3
12.5
25
50
100
Time (hours)

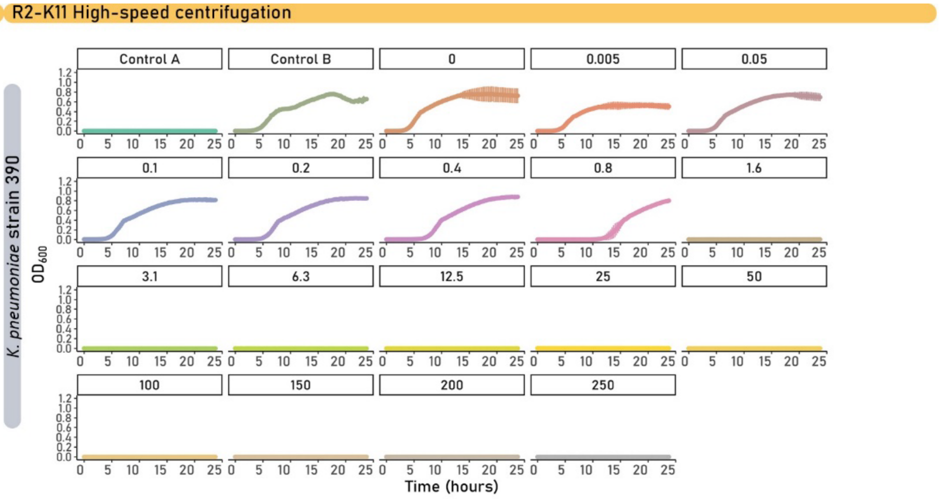
R2-K11 High-speed centrifugation
K. pneumoniae strain 390
OD600
Control A
Control B
0
0.005
0.05
0.1
0.2
0.4
0.8
1.6
3.1
6.3
12.5
25
50
100
150
200
250
Time (hours)

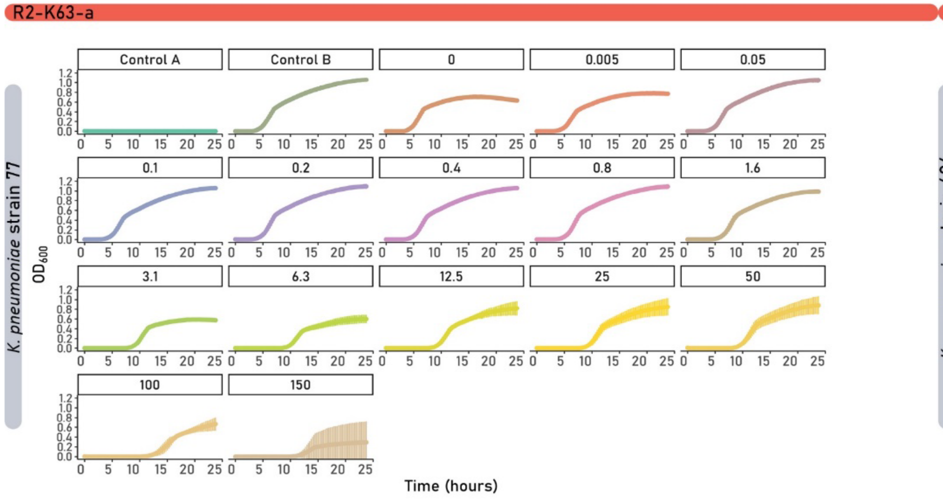
R2-K63-a
K. pneumoniae strain 77
OD600
Control A
Control B
0
0.005
0.05
0.1
0.2
0.4
0.8
1.6
3.1
6.3
12.5
25
50
100
150
Time (hours)

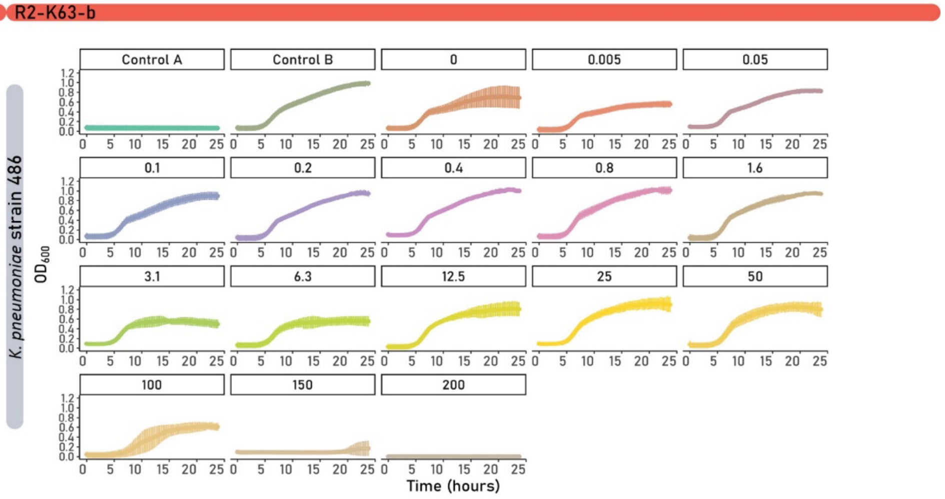
R2-K63-b
K. pneumoniae strain 486
OD600
Control A
Control B
0
0.005
0.05
0.1
0.2
0.4
0.8
1.6
3.1
6.3
12.5
25
50
100
150
200
Time (hours)

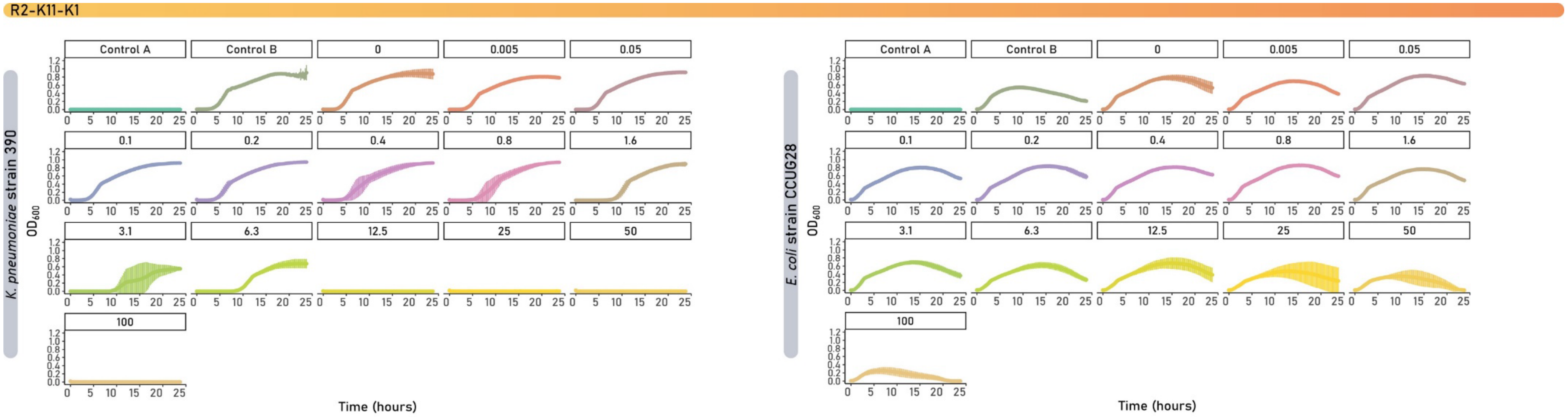

Supplementary Figure S5. 24-hour growth curves of the growth inhibition assay. The mean relative turbidity of the target strain mixed with different tailocins concentrations was monitored at 15-minute intervals over a 24-hour duration. Two additional controls were performed, one containing R2 tailocin but lacking the bacterial strain (Control A) and one containing a receptor-binding protein (RBP)-lacking mutant R2 tailocin particle (R2Δ*prf15*) instead of the engineered R2 tailocin of interest (Control B).

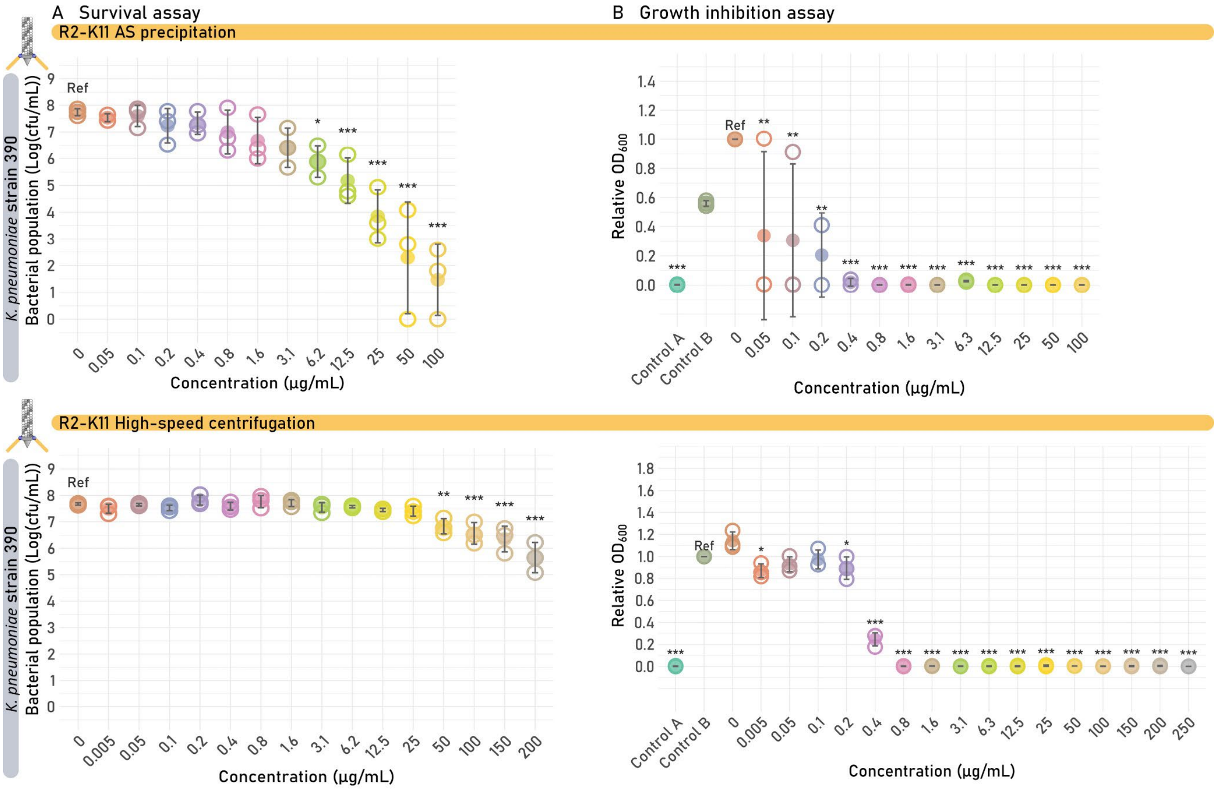

Supplementary Figure S6. Schematic overview and experimental results of R2 tailocin R2-K11 purified by two different methods, namely AS precipitation (upper graphs) and high-speed centrifugation (lower graphs). R2-K11 was tested on its susceptible *K. pneumoniae* target strain 390, which are indicated in vertical gray colored headings on the left side of the figure. Significant differences are shown using asterisks (*$p < 0.05$, **$p < 0.005$, ***$p < 0.001$). Reference (ref) indicates the value that was used as a reference for statistical comparison (untreated sample or control B). (**A**) Results of the survival assays. The plot showed the bacterial population in function of the concentration of the added R2-K11 tailocin. The value of each biological replicate is displayed using open circles and the mean values are shown as filled circles. Values of the untreated sample were used as reference for statistical analysis. (**B**) Results of the growth inhibition assay at 8 h are shown per R2-K11 tailocin purified with AS precipitation (upper graph) and high-speed centrifugation (lower graph). The relative OD_{600} of each biological replicate is displayed using open circles, and the mean relative OD_{600} is shown as full circles. Two additional controls were performed, one containing R2 tailocin but lacking the bacterial strain (Control A) and one containing an RBP-lacking mutant R2 tailocin particle (R2Δprf15) (Control B). Both controls were added at the same concentration as the highest available R2 tailocin concentration (200-250 µg/mL).

Supplementary Table S2. List of all bacterial strains and their origin used in this research.

Strain name	Origin	Additional information
***K. pneumoniae* strains**		
390	MIT[1]	Capsular serotype K11, susceptible to phage K11 (Ando et al., 2015)
31	IP[2]	Capsular serotype K31
77	UWr[3]	Capsular serotype K63, susceptible to phage KP34 and KP36
486	UWr[3]	Capsular serotype K63, susceptible to phage KP34 and KP36
***P. aeruginosa* strains**		
PAO1	KU[4], AvidBiotics	R2 tailocin producer (Williams et al., 2008)
PAO1 *Δprf15*	KU[4]	*P. aeruginosa* PAO1 lacking the R2 tailocin tail fiber gene *prf15* (Williams et al., 2008)
wtb/CF510	DTU[5]	Strain susceptible to PAO1 (Rau et al., 2012)
***E. coli* strains**		
5	ZHAW[6], UZH[7]	Serogroup O26
A11-2162	ZHAW[6], UZH[7]	Serotype O26:H31
NVH-848	NMBU[8]	Serotype O103:H25 (L'Abée-Lund et al., 2012)
4215/4	ZHAW[6], UZH[7]	Serogroup O103, susceptible to phage PAS7
P11-2315	ZHAW[6], UZH[7]	Serogroup O103
ECOR26	KU[4]	Serogroup O104 (Ochman & Selander, 1984)
ECOR27	KU[4]	Serogroup O104 (Ochman & Selander, 1984)
ECOR28	KU[4]	Serogroup O104 (Ochman & Selander, 1984)
10-1120	ZHAW[6], UZH[7]	Serogroup O111
P10-1119	ZHAW[6], UZH[7]	Serogroup O145
A11-1581	ZHAW[6], UZH[7]	Serotype O145:H34
A11-1675	ZHAW[6], UZH[7]	Serotype O146:H21, susceptible to phage PAS61
264	ZHAW[6], UZH[7]	Serogroup O157
332	ZHAW[6], UZH[7]	Serogroup O157
584	ZHAW[6], UZH[7]	Serogroup O157
777/1	ZHAW[6], UZH[7]	Serogroup O157
2905	ZHAW[6], UZH[7]	Serogroup O157
Sakai (stx-)	UoE[9]	Serotype O157:H7, susceptible to phage Tp10 (Dahan et al., 2004)
CAB1	UT Austin[10]	Serotype O18:K1:H7, susceptible to phage K1F (Bull et al., 2010)
TOP10	Invitrogen	F- *mcrA* Δ(*mrr-hsd*RMS-*mcr*BC) Φ80*lac*ZΔM15 Δ*lac*X74 *rec*A1 *ara*D139 Δ(*araleu*)7697 *gal*U *gal*K *rps*L (StrR) *end*A1 *nup*G
10G	LGC Genomics	
**Phages**		
Escherichia phage T7	MIT[1]	Host *E. coli* strain 10G
Escherichia phage vB_EcoP_PAS7	UGent, ZHAW[6]	Host *E. coli* strain 4215/4 (Pas et al., 2024)
Escherichia phage vB_EcoP_PAS61	UGent, ZHAW[6]	Host *E. coli* strain A11-1675 (Pas et al., 2024)
Escherichia O157 typing phage 10 (Tp10)	UoE[9]	Host *E. coli* strain Sakai (stx-) (Cowley et al., 2015)
Escherichia phage K1F	UT Austin[10]	Host *E. coli* strains CCUG28 and CAB1 (Bull et al., 2010)
Klebsiella phage K11	MIT[1]	Host *K. pneumoniae* strain KP390 (Ando et al., 2015)

Klebsiella phage KP34	UWr[3]	Host *K. pneumoniae* strains KP77 and KP486 (Drulis-Kawa et al., 2011; Maciejewska et al., 2023)
Klebsiella phage KP36	UWr[3]	Host *K. pneumoniae* strains KP77 and KP486 (Kęsik-Szeloch et al., 2013)
Klebsiella phage KP32	UWr[3]	Host *K. pneumoniae* strain KP271 and KP968 (Squeglia et al., 2020)

[1] Synthetic Biology Group, Massachusetts Institute of Technology (MIT), USA
[2] Institute Pasteur (IP), France
[3] Department of Pathogen Biology and Immunology, University of Wroclaw (UWr), Poland
[4] Department of Veterinary and Animal Sciences of the University of Copenhagen (KU; Denmark)
[5] Department of Biotechnology and Biomedicine, Technical University of Denmark (DTU; Denmark)
[6] Centre for Food Safety and Quality Management of the Zurich university of applied sciences (ZHAW, Switzerland))
[7] Institute for Food Safety and Hygiene of the University of Zurich (UZH)
[8] Department of Paraclinical Sciences of the Norwegian University of Life Sciences (NMBU)
[9] University of Edinburgh (UoE; Scotland)
[10] Department of Molecular Biosciences, University of Texas at Austin (MOL, UT Austin)

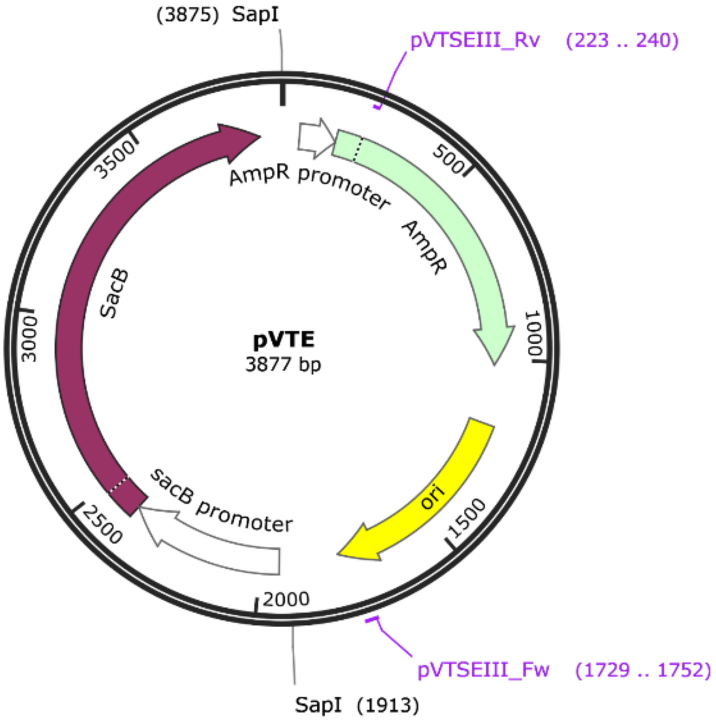

Supplementary Figure S7. Map of the pVTE cloning vector used in the VersaTile cloning step to construct tiles. Figure obtained using Snapgene v7.1.1.

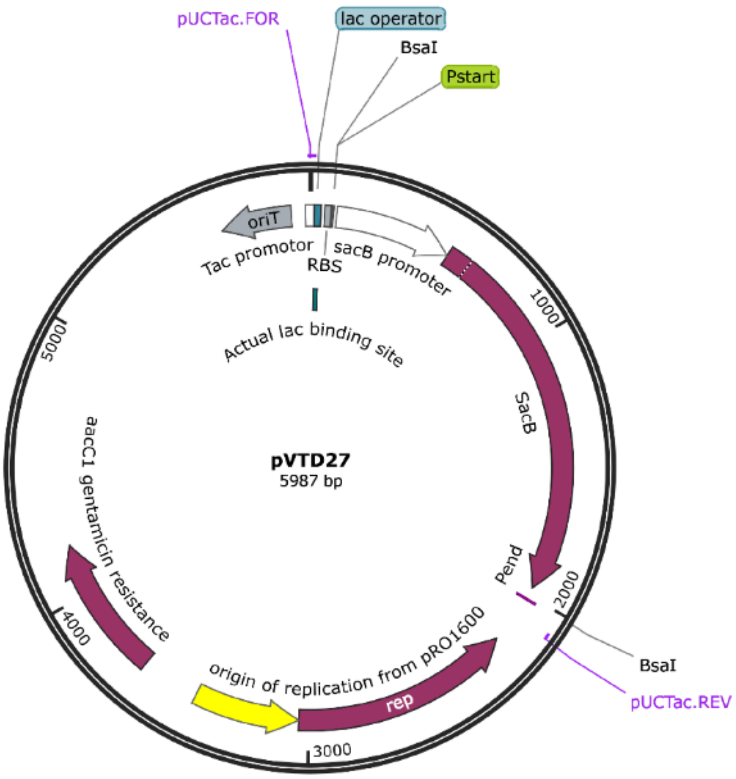

Supplementary Figure S8. Map of pVTD27 shuttle expression vector without a *lacI* repressor gene used for the VersaTile assembly reaction and expression of the receptor-binding protein in *Pseudomonas aeruginosa*. Figure obtained using Snapgene v7.1.1.

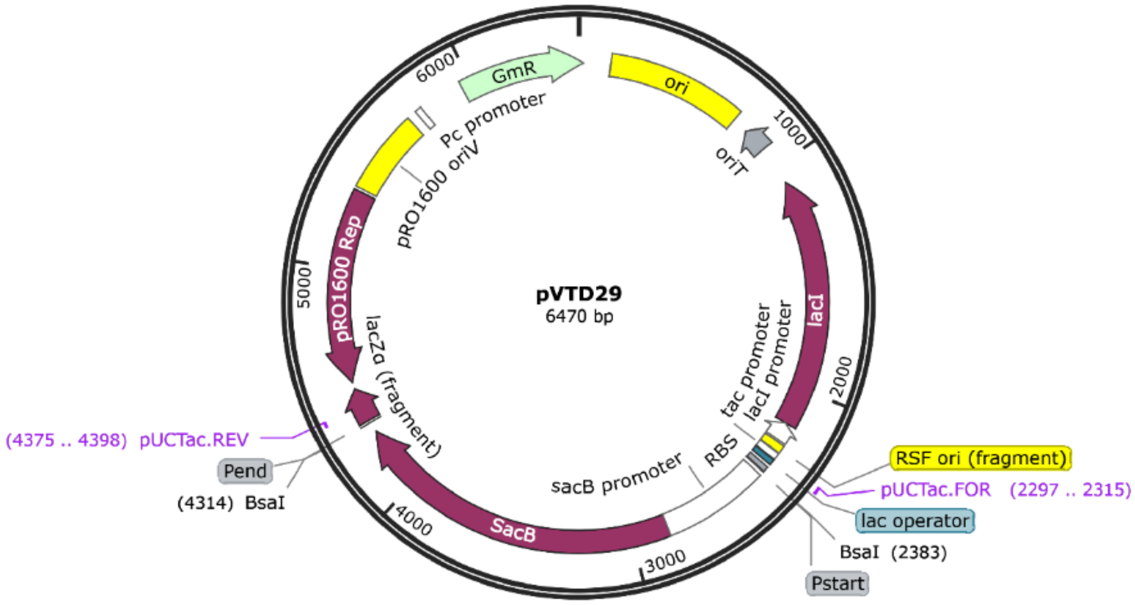

Supplementary Figure S9. Map of pVTD29 shuttle expression vector containing a *lacI* repressor gene used for the VersaTile assembly reaction and expression of the receptor-binding protein in *Pseudomonas aeruginosa.* Figure obtained using Snapgene v7.1.1.

Supplementary Table S3. Primers for tile preparation. Starting from the DNA ends, the following parts were attached: 1) a SapI recognition (underlined) and restriction site for cloning in the pVTE vector; 2) a BsaI recognition site (underlined) for VersaTile assembly; (3) and a position-specific tag of two amino acids defining their order in the final construct. The position-specific tags used for this engineered tailocin design were P_{start} (5’ ACCATG 3’) – P_{mid} (position tag middle 5’ GGTTCA 3’) for the anchor tile and P_{mid} - P_{end} (5’ GGTAAT 3’) for the RBD tile. These position tags are indicated in bold and restriction enzyme recognition sites (SapI and BsaI respectively) are underlined.

Tile name	Name Primer	Primer sequence	Target DNA	Target DNA Accession number	Amplified region (aa)
Anchor-DUF3751	BB2_DUF3751_BpiI.fw	GTGGAAGACGCTAGAGGTCTC**ACCATG**ACGACCAATACTCCGAAATACGG	PAprf15/16	NP_249311.1	1-163
	Rv_Anchor_P2P3_2	ATAGAAGACTAGCTTGGTCTC**TGAACC**CTTTTCCTTCACCCAGTCC			
RBP-PAprf15/16	BB2_DUF3751_BpiI.fw	GTGGAAGACGCTAGAGGTCTC**ACCATG**ACGACCAATACTCCGAAATACGG	PAprf15/16	NP_249311.1; NP_249312.1	1-691; 1-152
	Rv_RBP_P2P7_1	GTGGAAGACTAGCTTGGTCTC**ATTACC**TCCTCAGGGGGTGACATAACGTCTGAGC			
RBD-PAprf15/16	BB3_PEG0prf15-16_164.fw	GTGGAAGACGCTAGAGGTCTC**GGTTCA**GTCGCTGCCGATTTCAAGG	PAprf15/16	NP_249311.1; NP_249312.1	164-691; 1-152
	Rv_RBP_P2P7_1	GTGGAAGACTAGCTTGGTCTC**ATTACC**TCCTCAGGGGGTGACATAACGTCTGAGC			
RBD-K11	BB3_K11gp17E.Fw	GTGGCTCTTCAAGAGGTCTC**GGTTCA**GGCGTACTGCCGCTGTCTAATCTTCTG	K11gp17	YP_002003830.1	259-876
	BB3_K11gp17E.Rv	GTGGCTCTTCACTTGGTCTC**ATTACC**TCCTTATAAAACAAATGATGCTAATCTCGAGATG			
RBD-K11 (bivalent)	2E FOR Tile 2E FOR K11gp17E	GTGGCTCTTCAAGAGGTCTC**GGTTCA**GCAGATGACACCACGTCTCTCAAG	K11gp17	YP_002003830.1	179-876
	K11gp17 Enz875 P5 REV	GTGGCTCTTCACTTGGTCTC**TTCTCC**TTATAAAACAAATGATGCTAATCTCGAGATGTTC			
RBD-K31	fw_K11gp0043P3/P7	ATAGCTCTTCCAGAGGTCTC**GGTTCA**AACTACACCGACATGCAAGAG	K11gp0043	YP_002003831.1	2-597
	Rv_K11gp0043P3/P7	GTGGCTCTTCCCTTGGTCTC**ATTACC**TTAGTTAAGGAACAGCCTAACC			
RBD-KP63-a	KP34gp57P3P7_2/630	GTGGCTCTTCAAGAGGTCTC**GGTTCA**GCACTCACTAAACTAGTAGATGCAG	KP34gp57	YP_003347651.1	2-630
	BB3_KP34gp57.Rv	GTGGCTCTTCACTTGGTCTC**ATTACC**TCCCTAACCAGTGAGTTCAGATGGAGCAA			
RBD-KP63-b	BB3_KP36gp50.Fw	GTGGCTCTTCACTTGGTCTC**GGTTCA**AGCGCTGCGGCTGCTGCTGCGTCTGAAAATG	KP36gp50	YP_009226011.1	136-883
	BB3_KP36gp50.Rv	GTGGCTCTTCAAGAGGTCTC**ATTACC**TCCTTATGCCGTCAAATCTTCTAACTGAAGATTC			
RBD-K1	K1Fgp17_E_246_P3_FOR	ATAGCTCTTCAAGAGGTCTC**GGTTCA**GCTAAAGGGGATGGTGTCACTGATG	K1Fgp17	YP_424959.1	246-1065
	K1Fgp17_E_1065_P7	GTAGCTCTTCACTTGGTCTC**ATTACC**TCCTTACTTCTGTTCAAGAGCAGAAAGTCTGGC			
RBD-K1 (bivalent)	K1Fgp17_E_246_P6_FOR	ATAGCTCTTCAAGAGGTCTC**AGCGGA**GCTAAAGGGGATGGTGTCACTGATG	K1Fgp17	YP_424959.1	246-1065
	K1Fgp17_E_1065_P7_REV	GTAGCTCTTCACTTGGTCTC**ATTACC**TCCTTACTTCTGTTCAAGAGCAGAAAGTCTGGC			
RBD-O26	BB3_RBP_O26_119_Fw	GTGGCTCTTCCAGAGGTCTC**GGTTCA**CTCCTGAGCGAAGATCACTC	*E. coli* E2865 prophage tailspike	BBF60415.1	119-705
	BB3_RBP_O26_706_Rv	GTGGCTCTTCCCTTGGTCTC**ATTACC**TTAAAAGTTTCCCTCTGATAAG			
RBP-O103	BB3_RBP_7b_Fw	GTGGCTCTTCCAGAGGTCTC**GGTTCA**TCACTTGTAAGTCTAGTACGCGAC	PAS7 tailspike	WMX18832.1	2-480

	BB3_RBP_7b_Rv	GTG<u>GCTCTT</u>CCCTT<u>GGTCTC</u>**ATTACC**TTAAGCGAGTCTAGTGAATACCCCAC			
RBD-O104	BB3_RBP_O104_118_Fw	ATA<u>GCTCTT</u>CCAGA<u>GGTCTC</u>**GGTTC**ATTACAGGGGAGCGCAGGAGTC	*E. coli* LB226692 prophage tailspike	WP_000129924.1	118-659
	BB3_RBP_O104_660_Rv	GTG<u>GCTCTT</u>CCCTT<u>GGTCTC</u>**ATTACC**TTAATTGTGCTCCTTAGCAAAGCG			
RBD-O111	BB3_RBP_O111_Fw	GTG<u>GCTCTT</u>CCAGA<u>GGTCTC</u>**GGTTCA**CAATTAAGACAAGAGCTAGCGGGTC	*E. coli* 110512 prophage tailspike	WP_136721029.1	113-645
	BB3_RBP_O111_Rv	GTG<u>GCTCTT</u>CCCTT<u>GGTCTC</u>**ATTACC**CTAGCCATCATACCAAGCATTGTTAAG			
RBD-O145	BB3_RBP_O145_118_Fw	GTG<u>GCTCTT</u>CCAGA<u>GGTCTC</u>**GGTTCA**TTCCCTCAGCAACTTTCTCAATTAGG	*E. coli* FHI58 prophage tailspike	LM995999.1	118-675
	BB3_RBP_O145_676_Rv	GTG<u>GCTCTT</u>CCCTT<u>GGTCTC</u>**ATTACC**CTATTCTGAAATATATGATCCG			
RBD-O146	BB3_RBP_61_Fw	GTG<u>GCTCTT</u>CCAGA<u>GGTCTC</u>**GGTTCA**GCAACAAAGAATTATGCTGATGAGC	PAS61 tailspike	WMX19002.1	224-745
	BB3_RBP_61_Rv	GTG<u>GCTCTT</u>CCCTT<u>GGTCTC</u>**ATTACC**TCAGTTAGCCAGCGATACAGAC			
RBD-O157	BB3_RBP_Tp10_Fw	GTG<u>GCTCTT</u>CCAGA<u>GGTCTC</u>**GGTTCA**GCTACAAAAAAATATGTTGATGGGGTCGC	Tp10 tailspike	AKE46458.1	216-875
	BB3_RBP_Tp10_Rv	GTG<u>GCTCTT</u>CCCTT<u>GGTCTC</u>**ATTACC**TTAAATATTTCCGTTCAGCGTGCGG			
CP-KP32 (bivalent)	CP_K32_F	GTG<u>GAAGAC</u>GCTAGA<u>GGTCTC</u>**GGAGAA**CCTCATGTTAGACAATTTCAATCAG	KP32 conserved pepide (CP)	NC 013647.1	1-35
	CP_KP32_R	ATA<u>GAAGAC</u>GCGCTT<u>GGTCTC</u>**TCCGCT**GAAGGACTCAAGGCGCGGTAATG			

Supplementary Table S4. Components and conditions for tile sequence amplification for the VersaTile cloning step using Phusion polymerase. Annealing temperature 1 (TA1) represented the optimal TA only for the matching parts of both the forward and the reverse primer. This temperature was used in the first ten cycles to ensure efficient primer annealing to the template. The Thermo Fisher Scientific TA calculator tool was used to calculate the optimal annealing temperature.

Ingredients	Volume
5x High-Fidelity buffer (Thermo Fisher)	10 µL
10 mM dNTPs (Thermo Fisher)	1 µL
Forward primer (10 µM)	2.5 µL
Reverse primer (10 µM)	2.5 µL
Template DNA	50-100 ng
Phusion DNA polymerase (2 U/µL) (Thermo Fisher)	0.5 µL
Ultrapure water	variable
Total volume	50 µL

Conditions	Temperature (°C)	Time	Cycles
Initial denaturation	98	30 s	1
Denaturation	98	10 s	10
Annealing	TA1	25 s	
Extension	72	30 s/kb (<30s)	
Denaturation	98	10 s	20
Annealing	72	25 s	
Extension	72	30 s/kb (≥ 30 s)	
Final extension	72	10 min	1

Supplementary Table S5. Components and conditions for the restriction-ligation reaction of the VersaTile cloning step.

Ingredients	Volume
Buffer T4 ligase (Thermo Fisher)	2 µL
T4 DNA Ligase (5 U/µL) (Thermo Fisher)	3 µL
SapI (5 U/µL) (Thermo Fisher)	1 µL
pVTE	25 nM
Tile fragment	46 nM
Ultrapure water	variable
Total volume	20 µL

Conditions	Temperature (°C)	Time	Cycles
Restriction	37	2 min	30
Ligation	22	3 min	
Ligase inactivation	50	5 min	1
Type IIs inactivation	65	20 min	1

Supplementary Table S6. Components and conditions for the restriction-ligation reaction of the VersaTile assembly step.

Ingredients	Volume
pVTD27/pVTD29 (25 nM)	1 µL
Tile (46 nM)	1 µL/Tile
10X ligation buffer (Thermo Fisher)	2 µL
BsaI (10 U/µL) (Thermo Fisher)	1 µL
T4 DNA ligase (5 U/µL) (Thermo Fisher)	3 µL
Ultrapure water	variable
Total volume	20 µL

Conditions	Temperature (°C)	Time	Cycles
Restriction	37	5 min	25
Ligation	22	5 min	
Ligase inactivation	50	5 min	1
BsaI inactivation	80	5 min	1

Supplementary Table S7. Overview of the different wild-type and engineered R2 tailocins that were produced in this work. The receptor-binding protein (RBP) expression method, the possible nucleotide junction between anchor and receptor-binding domain (RBD), the method of purification and the expression vector that was used are indicated per construct.

Construct name	RBD origin	RBD origin accession	Expression RBP	Interdomain junction (nt)	Purification method	Expression vector
R2-WT	*P. aeruginosa* strain PAO1	NC_002516.2	In genome	/	Both	/
R2-WT-trans	*P. aeruginosa* strain PAO1	NC_002516.2	*In trans*	/	Both	pVTD29
R2-WT-VT	*P. aeruginosa* strain PAO1	NC_002516.2	*In trans*	GGTTCA	Both	pVTD29
R2*Δprf15*	*P. aeruginosa* strain PAO1*Δprf15*	/	/	/	Both	/
R2-O26	*E. coli* strain RM10386 prophage	AP018808.1	*In trans*	GGTTCA	High-speed centrifugation	pVTD29
R2-O103-a	Escherichia phage vB_EcoP_PAS7	OQ921331.1	*In trans*	GGTTCA	High-speed centrifugation	pVTD29
R2-O103-b	Escherichia phage vB_EcoP_PAS7	OQ921331.1	*In trans*	CTTGGTTCA	High-speed centrifugation	pVTD27
R2-O104	*E. coli* strain LB226692 prophage	NZ_CP024992.1	*In trans*	GGTTCA	High-speed centrifugation	pVTD29
R2-O111	*E. coli* strain 110512 prophage	NZ_AP019761.1	*In trans*	GGTTCA	High-speed centrifugation	pVTD29
R2-O145-a	*E. coli* strain FHI58 prophage	LM995999.1	*In trans*	GGTTCA	High-speed centrifugation	pVTD29
R2-O145-b	*E. coli* strain FHI58 prophage	LM995999.1	*In trans*	CTTGGTTCA	High-speed centrifugation	pVTD27
R2-O146	Escherichia phage vB_EcoP_PAS61	OQ921333.1	*In trans*	GGTTCA	High-speed centrifugation	pVTD29
R2-O157	Escherichia phage O157 typing phage 10 (Tp10)	KP869108.1	*In trans*	GGTTCA	High-speed centrifugation	pVTD29
R2-K1	Escherichia phage K1F	NC_007636.1	*In trans*	GGTTCA	High-speed centrifugation	pVTD29
R2-K11	Klebsiella phage K11	NC_011043.1	*In trans*	GGTTCA	Both	pVTD29
R2-K31	Klebsiella phage K11	NC_011043.1	*In trans*	GGTTCA	AS precipitation	pVTD29
R2-K63-a	Klebsiella phage KP34	NC_013649.2	*In trans*	GGTTCA	High-speed centrifugation	pVTD29
R2-K63-b	Klebsiella phage KP36	NC_029099.1	*In trans*	GGTTCA	High-speed centrifugation	pVTD29
R2-K11-K1	Klebsiella phage K11/ Escherichia phage K1F	NC_011043.1 /NC_007636.1	*In trans*	GGTTCA	High-speed centrifugation	pVTD29

VT: VersaTile; AS: ammonium sulfate

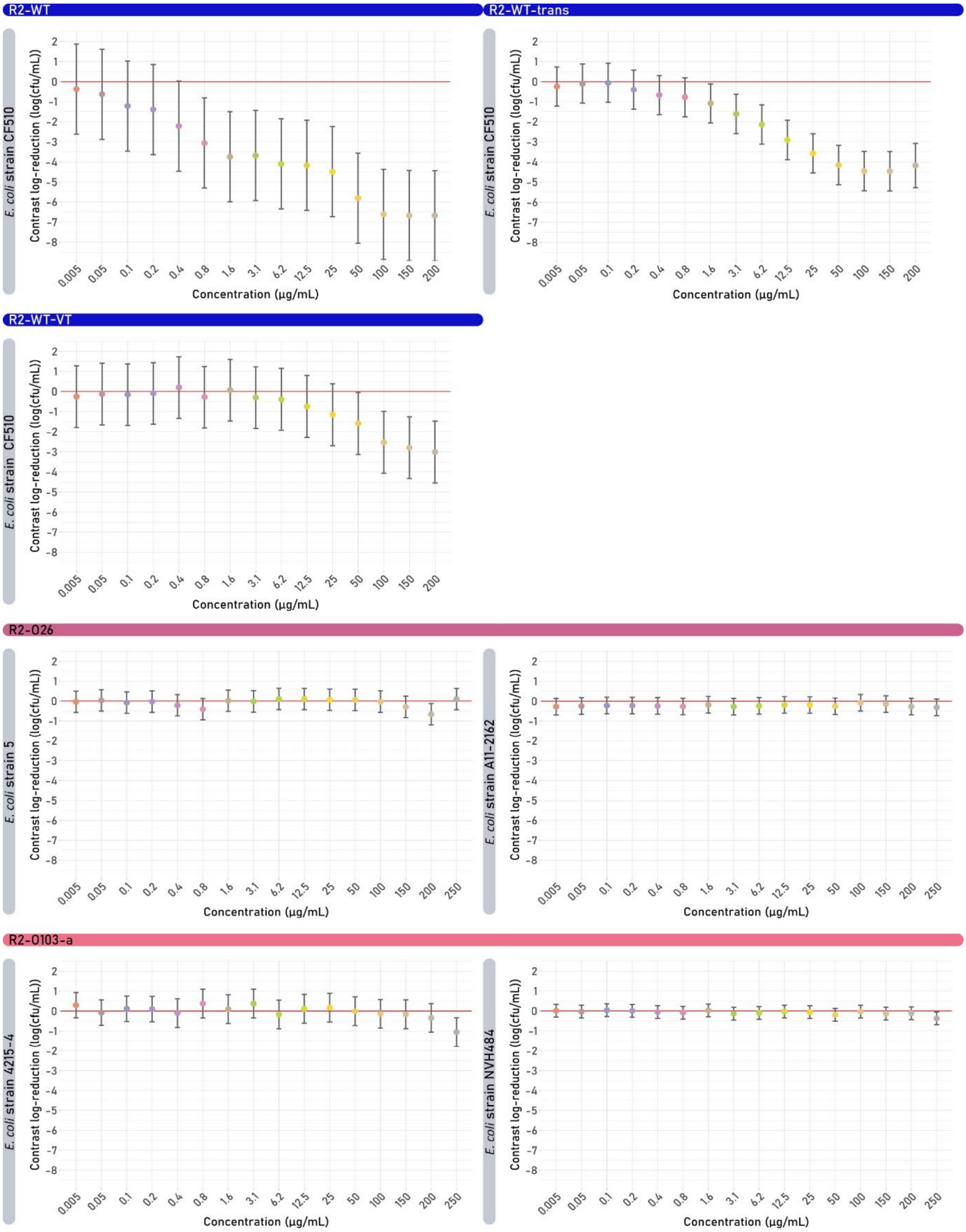
R2-WT
R2-WT-trans
R2-WT-VT
R2-O26
R2-O103-a
E. coli strain CF510
E. coli strain 5
E. coli strain A11-2162
E. coli strain 4215-4
E. coli strain NVH484
Contrast log-reduction (log(cfu/mL))
Concentration (µg/mL)
0.005
0.05
0.1
0.2
0.4
0.8
1.6
3.1
6.2
12.5
25
50
100
150
200
250

R2-0103-b

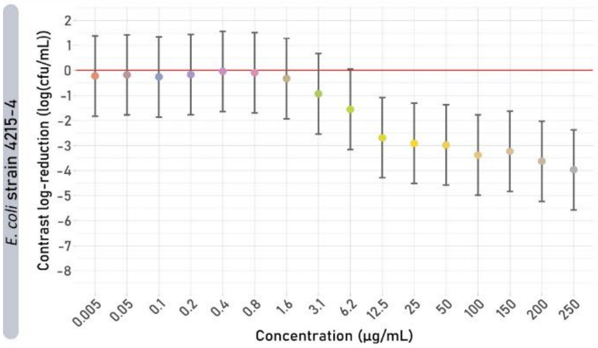

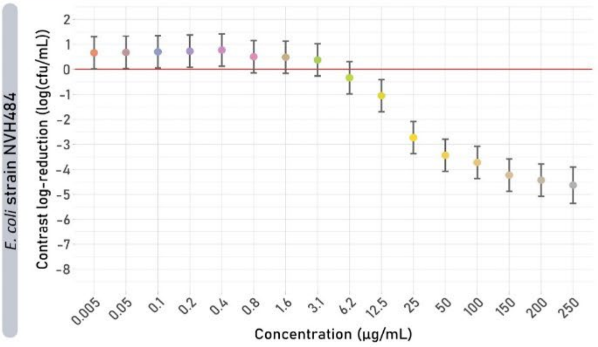

R2-0104

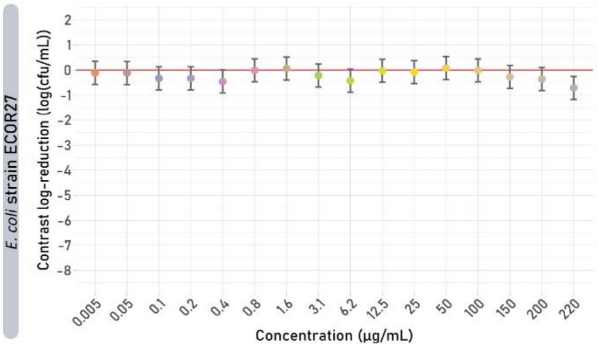

R2-0111

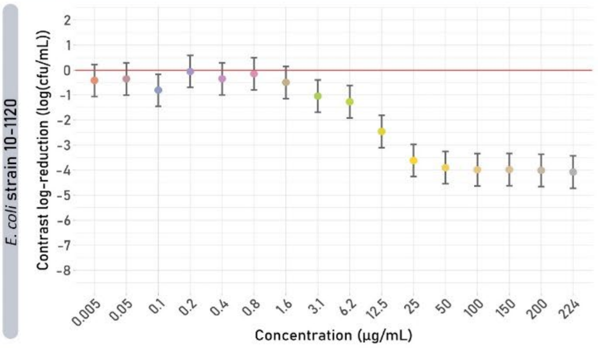

R2-0145-a

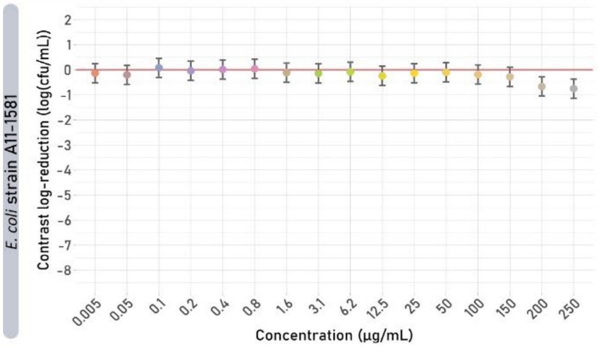

R2-0145-b

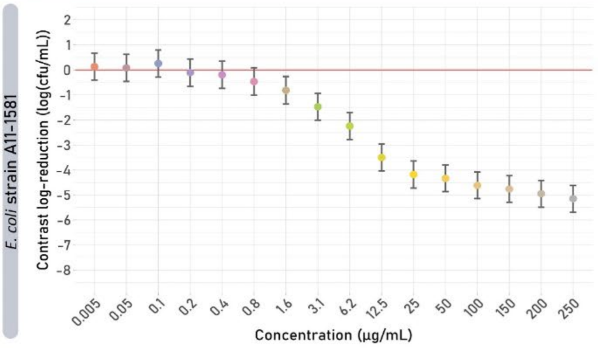

R2-0146

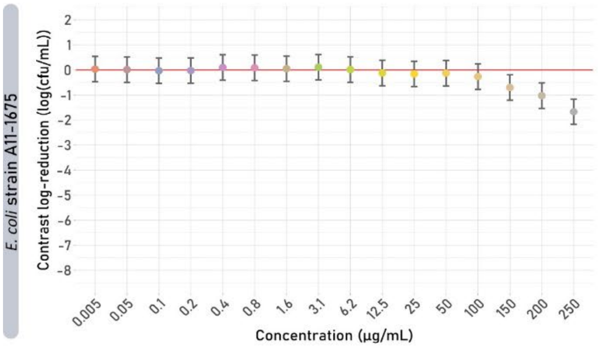

R2-0157

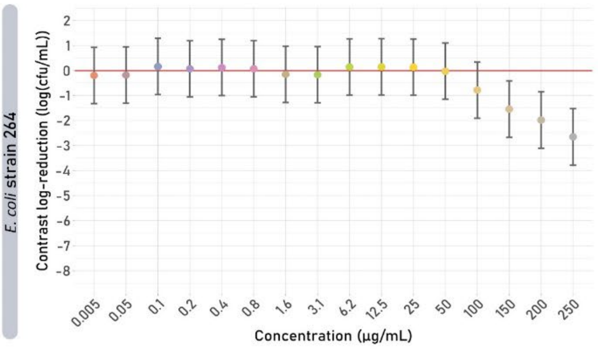

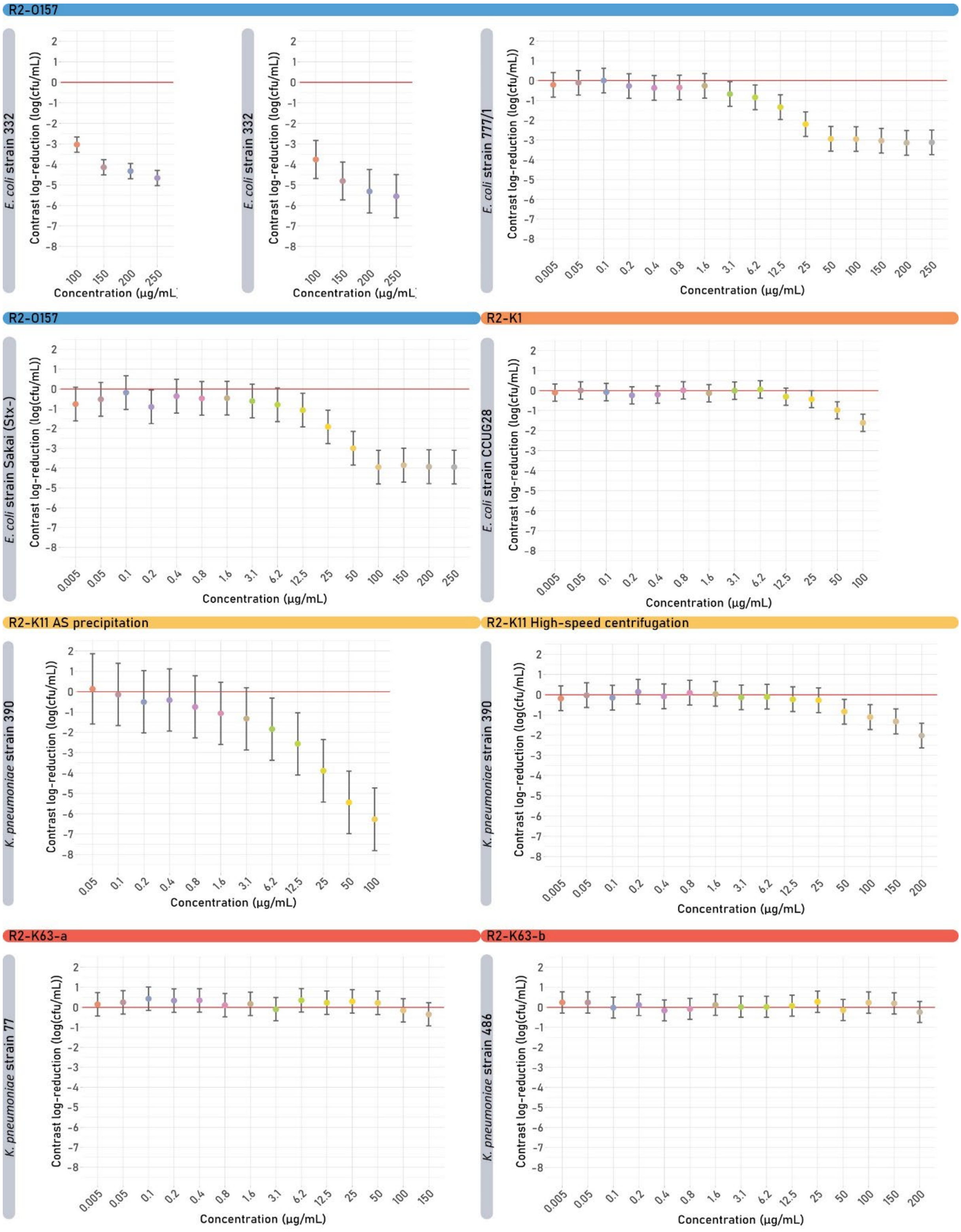
R2-O157
E. coli strain 332
Contrast log-reduction (log(cfu/mL))
Concentration (µg/mL)
E. coli strain 777/1
R2-O157
E. coli strain Sakai (Stx-)
R2-K1
E. coli strain CCUG28
R2-K11 AS precipitation
K. pneumoniae strain 390
R2-K11 High-speed centrifugation
K. pneumoniae strain 390
R2-K63-a
K. pneumoniae strain 77
R2-K63-b
K. pneumoniae strain 486

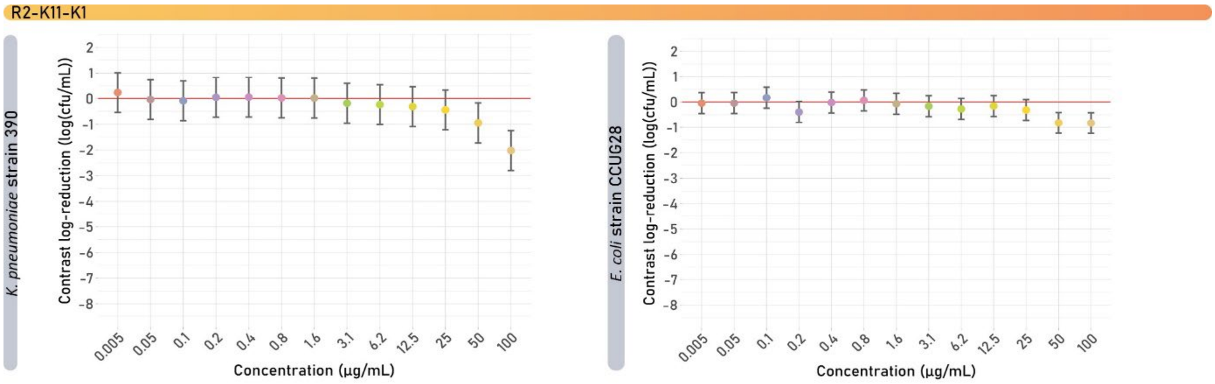

Supplementary Figure S10. Statistical analysis of the survival assay. A linear mixed model with random intercept per biological replicate was fitted to the data. The bacterial log-reduction compared to the zero concentration, or the contrast in short, was plotted in function of the concentration of the produced R2 tailocin with joint confidence intervals. All concentrations for which the confidence interval lies below the zero line indicate a significant bacterial log-reduction at that concentration.
